# Plasmonic and Photothermal Properties of Silica-Capped Gold Nanoparticle Aggregates

**DOI:** 10.1021/acs.jpcc.3c07536

**Published:** 2023-12-12

**Authors:** Jodie Fergusson, Gregory Q. Wallace, Sian Sloan-Dennison, Ruairí Carland, Neil C. Shand, Duncan Graham, Karen Faulds

**Affiliations:** † Centre for Nanometrology, Department of Pure and Applied Chemistry, Technology and Innovation Centre, 99 George Street, Glasgow G1 1RD, U.K.; ‡ Defence Science and Technology Laboratory, Porton Down, Salisbury SP4 0JQ, U.K.

## Abstract

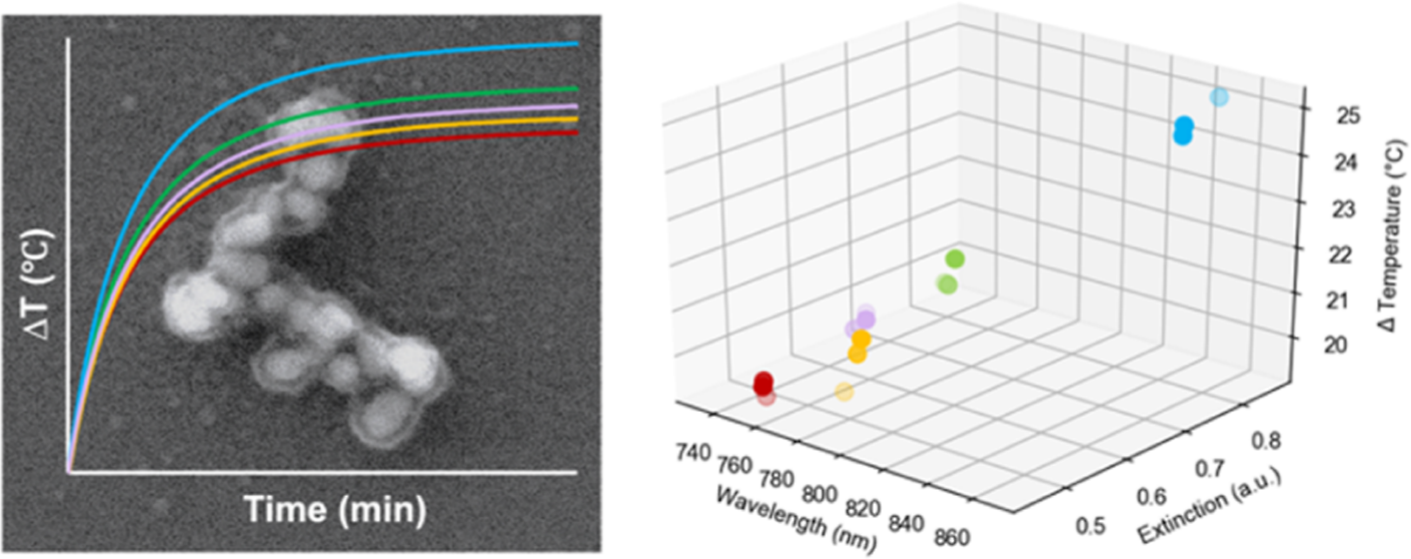

Owing to their biocompatibility, gold nanoparticles have many applications in healthcare, notably for targeted drug delivery and the photothermal therapy of tumors. The addition of a silica shell to the nanoparticles can help to minimize the aggregation of the nanoparticles upon exposure to harsh environments and protect any Raman reporters adsorbed onto the metal surface. Here, we report the effects of the addition of a silica shell on the photothermal properties of a series of gold nanostructures, including gold nanoparticle aggregates. The presence of a Raman reporter at the surface of the gold nanoparticles also allows the structures to be evaluated by surface-enhanced Raman scattering (SERS). In this work, we explore the relationship between the degree of aggregation and the position and the extinction of the near-infrared plasmon on the observed SERS intensity and in the increase in bulk temperature upon near-infrared excitation. By tailoring the concentration of the silane and the thickness of the silica shell, it is possible to improve the photothermal heating capabilities of the structures without sacrificing the SERS intensity or changing the optical properties of the gold nanoparticle aggregates.

## Introduction

Metallic nanostructures support plasmonic properties that can span from the UV through to the infrared region of the electromagnetic spectrum.^1^ These optical properties, such as the presence of a localized surface plasmon resonance (LSPR), can be readily tuned by changing the material composition,^2^ the refractive index,^3^ and their opto-geometric structure.^4^ For example, anisotropic nanostructures, such as nanorods, support both a transverse and a longitudinal LSPR.^5^ The presence of the LSPR has allowed for metallic nanostructures to see great utility in areas of vibrational spectroscopy,^6^ catalysis,^7^ and therapeutics.^8^ Developing nanostructures with near-infrared (NIR) compatibility is especially desirable for applications involving biological materials as NIR wavelengths penetrate deeper into tissue compared to visible light as there are fewer losses from absorption and scattering.^9,10^

When the nanostructures are irradiated with light, the incident energy is dissipated by radiative and nonradiative pathways.^7^ Of the nonradiative pathways, the generation of heat,^11^ known as plasmonic heating, and subsequent transfer of the heat from the metal nanoparticles to the surrounding environment, is of particular interest. This generated heat can be used to trigger the release of a drug found at the surface of the nanoparticles^12^ and/or create a localized hyperthermic environment. Cancer cells are known to have low resistance to heat due to their poor blood and oxygen flow,^13^ therefore, as the temperature of the local environment increases, more of the cancerous cells will undergo cell death. The amount of cell death and the mechanisms by which it occurs are heavily dependent on the achieved temperature.^14^ In general, temperatures in excess of 40 °C are required to eliminate tumors.^15^

Given the relationship between increased temperature and amount of cell death, there is much interest in developing plasmonic nanostructures with maximized photothermal heating capabilities. Structures that have shown promise for photothermal applications include gold nanoshells,^16^ nanostars,^17^ nanorods,^18^ and hybrid structures such as “nanomatryoshkas”.^19^

In addition to isolated structures, nanoparticle aggregates have shown potential in photothermal therapy as they often possess LSPRs in the NIR.^20^ Aggregation of the gold nanoparticles (AuNPs) is induced by exposure to a specific trigger and can be performed either ex situ or in situ. Ex situ aggregation is simpler and provides greater control over the aggregate size. As demonstrated by Wang et al., the degree of aggregation of AuNPs can be semicontrolled by varying the concentration of added NaCl.^21^ Similarly, the work of Sun and co-workers showed that phosphate-buffered saline, cell media, and interstitial fluid could be used to aggregate AuNPs, with those aggregated via phosphate-buffered saline showing a temperature increase of 26.3 °C within 5 min when irradiated using 808 nm laser light.^22^ Other examples of aggregating agents that have been used to prepare photothermally relevant plasmonic aggregates include bovine serum albumin^23^ and dimethyl sulfoxide.^24^ Alternatively, aggregation can also be triggered in situ, and often this involves utilizing the lower pH value of cancerous cells in comparison to healthy cells, to induce preferential aggregation within the more acidic cancer cells. Work by Nam et al.,^25^ demonstrated that AuNPs functionalized with a surface molecule which became positively charged in acidic conditions (pH = 5.5) and induced aggregation were more effective at inducing cell mortality when irradiated using a 660 nm laser compared with citrate-capped AuNPs. This same approach was then extended to the endosomes of mesenchymal stem cells as they also have a local acidic environment.^26^ Another option is to use single-stranded DNA and cytochrome *c*, as demonstrated by Park et al., where the surface charge of cytochrome *c* became positive under acidic conditions and caused aggregation.^27^ Other methods of inducing aggregation within cells include functionalizing the nanoparticle surface with a peptide which is sensitive to alkaline phosphatase, an enzyme found in tumors.^28^ In general, an in vivo approach derived from a cell-specific target is more selective as the photothermal activity is achieved only when the nanoparticles are inside the tumor environment and therefore less likely to cause damage to surrounding healthy cells.

However, one of the main challenges in using aggregated nanoparticles is that this process can often be uncontrolled and result in overaggregation. This is especially true for in vivo aggregation, where aggregates more than 400 nm in diameter have been reported within cells.^26^ The addition of a protective shell can be utilized to minimize overaggregation and promote stability. Adding such a shell is relatively straightforward during ex vivo synthesis. Polymers such as polyethylene glycol or polyvinylpyrrolidone or dielectric materials, notably silica, have all shown promise. In the case of silica, it was recently demonstrated that the presence of a silica shell around spherical AuNPs did not significantly lower the temperatures achieved during the photothermal irradiation with a 532 nm laser, compared to uncoated AuNPs.^29^

It is also desirable to be able to track photothermal agents in vivo, to monitor that first, they have reached their target, and second, to ensure that they are expelled from the body in full. One method of achieving this is by using surface-enhanced Raman scattering (SERS). In the context of cells and tissues, the ability to readily functionalize the nanoparticle surface allows for nanoparticles to be locally delivered to specific cells, such as cancer cells^30^ while the presence of a Raman reporter enables tracking of the nanoparticles.^31,32^ Nanoparticle aggregates are known to give extremely intense electromagnetic enhancement and thus strong SERS signals.^20^ Structures such as nanorods and nanostars can also achieve the necessary combination of SERS compatibility and heat generation. However, these structures often revert to more thermodynamically stable spheres upon photothermal irradiation,^33^ thus dampening both the SERS intensity and future photothermal heating. Encapsulation of the nanostructure within a silica shell can help in improving photothermal stability while also protecting the Raman reporter from the biological environment and dissociation from the metal surface.^34^ As a result, such structures become effective for targeted photothermal drug release and nanoparticle tracking within biological systems.^35^

Although examples of (i) aggregated gold nanostructures for photothermal heating have previously been reported in the literature, along with (ii) the effects of a protective silica shell on temperature and (iii) the dual functionality of heating combined with SERS, the combination of these three aspects, to the best of our knowledge, have not yet been explored. In this work, we compare the photothermal capabilities of AuNPs, gold-core–shell-isolated nanoparticles (SHINs), and silica-capped AuNP aggregates. As opposed to using any of the previously described aggregation means, Raman reporter-induced aggregation was used.^36^ These aggregates not only exhibited a superior photothermal response to the other structures but also their optical properties remain undisturbed following prolonged irradiation, including a high degree of stability. By systematically investigating aspects of the synthesis of the aggregates, such as the degree of aggregation and the thickness of the silica shell, we demonstrate the relationship between the optical and physical properties of the aggregates and their heating capability. This is a significant step forward in the field of plasmonics and photothermal heating, especially for the ex vivo preparation of plasmonic aggregates, while also providing valuable experimental insights into how to design such substrates to maximize their heating capabilities and stability.

## Materials and Methods

### Chemicals

All chemicals were purchased from Sigma-Aldrich.

### AuNP Synthesis

AuNPs were prepared using a refined Turkevich synthesis, on a 20 L scale.^37^ 2.42 g NaAuCl_4_–H_2_O was added to 20 L doubly deionized distilled H_2_O with stirring and heated to 100 °C. After 90 min of heating, 2.3 g of sodium citrate in 300 mL H_2_O was added, yielding a mole ratio of 1:1.286 for NaAuCl_4_–H_2_O to citrate, respectively. The mixture was left to cool overnight with stirring.

### Synthesis of Shell-Isolated Nanoparticles

The synthesis of SHINs was adapted from the protocol originally reported by Li et al.^38^ 50 mL of previously synthesized AuNP were added to a round-bottom flask with stirring. To this, 150 μL of 100 μM 1,2-bis(4-pyridyl)ethylene (BPE), made up as a 10 mM stock solution in ethanol and subsequently diluted with water, was added to give a final concentration of 300 nM. The SERS signal was checked using a hand-held 785 nm spectrometer to ensure a sufficient signal had been achieved. To the now functionalized AuNPs, 75 μL of 3-aminopropyltrimethoxysilane (APTMS) (0.02%) and 750 μL of sodium silicate (20%) were rapidly added, to grow the silica shell. The mixture was heated to 90 °C for 30 min and cooled overnight with stirring.

### Synthesis of Silica-Capped Aggregates

Inspired by the synthesis of the SHINs, 45 mL of previously synthesized AuNPs was added to a round-bottomed flask with stirring. To this, 1 mL of BPE at a concentration of 23 μM was added to give a final concentration of 500 nM and induce aggregation. 75 μL of 1% APTES and 50 μL of sodium silicate were rapidly added to further promote aggregation and eventually grow the silica shell that would then arrest the aggregation. The mixture was heated at 90 °C for 30 min and left to cool overnight with stirring.

### Adjusting the APTES Concentration

Five batches of BPE SiO_2_ aggregates were prepared as previously discussed using the same batch of AuNPs, with the only difference being increasing the aliquots of 1% APTES used: 30, 50, 75, 80, and 100 μL to give final APTES concentrations of 6.5, 10.9, 16.2, 17.3, and 21.7 μM, respectively. All other concentrations of reagents and reaction steps were as previously stated for aggregate synthesis.

### Increasing the Silica Shell Thickness

30 mL of previously prepared SHINs and aggregates were centrifuged at 6000 rpm for 20 min, and 20 mL of supernatant was removed leaving 10 mL of the concentrated sample. The concentrated solution was then added to a round-bottom flask. To each solution, 1.7 mL of ethanol (EtOH) and 400 μL of ammonium hydroxide (NH_4_OH) were added and allowed to stir for 5 min before the addition of 16.6 μL of tetraethyl orthosilicate (TEOS). The mixtures were stirred overnight to allow growth of the silica shell.

### Sample Characterization

Samples were characterized before and after heating using extinction spectroscopy, dynamic light scattering (DLS), and SERS. Extinction measurements were carried out by using a Cary 60 UV-vis spectrometer, scanning from 300 to 1100 nm at a scan rate of 600 nm/min. DLS measurements were carried out using a Malvern Nanoseries Zetasizer, using an SOP optimized for the refractive index of gold. SERS measurements were carried out using a Snowy CBex hand-held spectrometer at an excitation wavelength of 785 nm. For all SERS measurements, the lowest laser power setting (10 mW) and an acquisition time of 0.1 s was used. SERS spectra were baseline corrected by using MATLAB (Version 2022b) and plotted in Excel. All scans were carried out in triplicate and averaged. For extinction and SERS measurements, samples were prepared to an optical density of 1 based on the visible plasmon at ∼540 nm for a volume of 500 μL; DLS measurements were carried out undiluted.

### Imaging

Scanning electron microscopy (SEM) imaging was carried out using an FEI Quanta 250 FEG scanning electron microscope from Oxford Instruments, with a voltage of 30 kV and a spot size of 3.5. Samples were prepared by spotting 1 μL of sample onto a plasma-cleaned silica wafer and allowing it to dry overnight. Transmission electron microscopy (TEM) imaging was carried out at the University of Glasgow using a JEOL JEM-1400Flash at an 80 kV accelerating voltage. Samples were prepared by drop-casting 1 μL of sample onto 200 mesh carbon-coated copper TEM grids. Following TEM, silica shell thickness was measured using ImageJ by taking 25 measurements across each sample and calculating an average.

### Photothermal Heating of Samples

A custom-built photothermal setup was used, where a 785 nm laser beam (maximum laser power of 300 mW at the source) was directed toward the sample holder by a mirror and lens. A welded tip fiberglass thermocouple (TCI Direct, 401-941) was inserted directly into the colloidal solution outside of direct contact with the beam path. A TC-08 thermocouple data logger (Pico Technology Ltd.) and PicoLog software was used to record the temperature at 1 s intervals. For all heating experiments, the optical density of each sample was adjusted to 1 to allow for an accurate comparison, using the plasmon at ∼540 nm as this was the most intense for all samples. 500 μL of sample was placed in a glass vial in the path of the laser beam, with the temperature probe positioned adjacent to the beam. All samples were heated in triplicate, and an average was taken. The laser power was measured by using a ThorLabs power meter (PM100D) equipped with a S130C 400–100 nm sensor.

## Results and Discussion

### Optical Properties and Plasmonic Heating of Spherical AuNP-Derived Structures

The preparation of the spherical AuNP aggregates is based on the synthesis of SHINs.^39^ In general, the synthesis of such SHINs and aggregates can be summarized into three or four steps: (i) synthesis of spherical AuNPs, (ii) addition of a Raman reporter, (iii) addition of an aminosilane to render the surface vitreophilic and enable the formation of a thin silica shell to minimize over-aggregation, and (iv) optionally increasing the thickness of the silica shell using a modified Stöber process.^40^ The noticeable difference between the SHINs and the aggregates is that a larger final concentration of the Raman reporter is needed to induce the aggregation [500 nM (1,2-bis(4-pyridyl)ethylene) BPE] for the aggregates and 300 nM BPE for the SHINs. Additionally, a higher concentration of aminosilane was used in the synthesis of the aggregates, resulting in more destabilization of surface citrate, resulting in further aggregation. For the best possible comparison, the same batch of AuNPs was used in the preparation of both the SHINs and the aggregates used in this section. The use of the Raman reporter as the aggregating agent is particularly attractive in the ex vivo synthesis of plasmonic aggregates when compared to other approaches. If the aggregates are preformed and subsequently functionalized, then it is necessary for the reporter to diffuse into the nanoscale junction of strong electromagnetic field enhancement or hotspot. Alternatively, the AuNPs could be functionalized with the reporter, as with the SHINs, and then aggregated by an external trigger, such as NaCl. However, there is once again a concern if the reporter would be present within the hotspot as well as any desorption of the reporter by the aggregating agent. By utilizing the Raman reporter as the aggregating agent, there exists a greater likelihood that the reporter molecule will be within the hotspot, with the only source of desorption being from the growth of the encapsulating shell.

**Figure 1:**
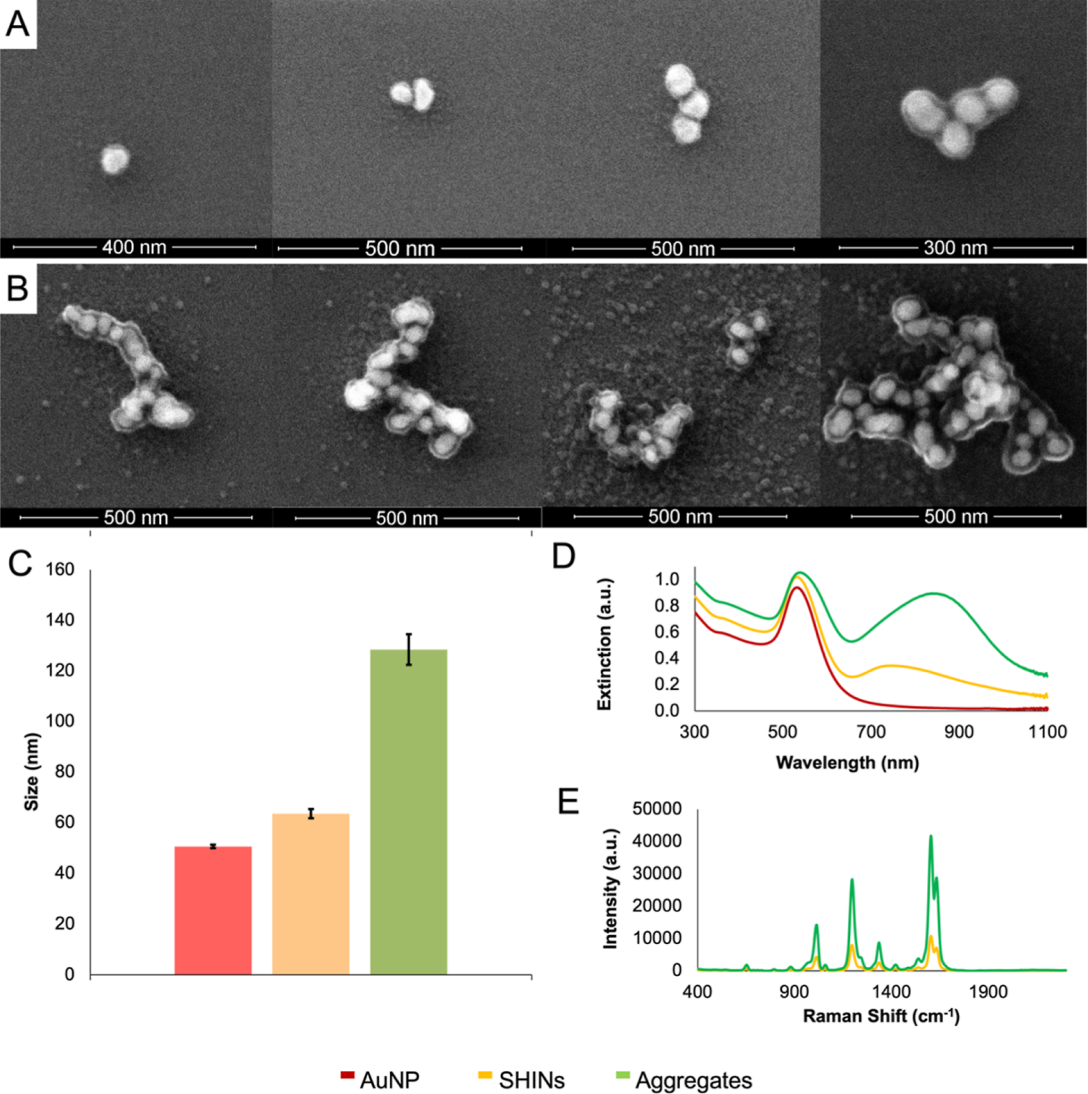
SEM images of a variety of (A) SHINs functionalized with BPE, and (B) silica-capped BPE-functionalized AuNP aggregates. (C) DLS measurements for the AuNP, SHINs, and aggregates. (D) Extinction spectra for the three nanostructures, collected using a Cary60 UV–vis spectrophotometer scanning from 300 to 1100 nm at a medium scanning rate of 600 nm/min. (E) SERS spectra of SHINs and aggregates, collected using a hand-held Snowy Range Instruments CBEx spectrometer at an excitation wavelength of 785 nm, a laser power of 10 mW at the sample, and an acquisition time of 0.1 s. Following collection, spectra were baseline corrected using MATLAB (Version 2022b) and plotted in Excel. For characterization with extinction spectroscopy and SERS, samples were adjusted to an optical density of 1 for a volume of 500 μL. DLS measurements were performed using the samples as prepared.

The SEM images in Figure 1A show that the SHINs comprise a mixture of single-core structures and smaller aggregates composed of a few AuNPs. The addition of both the BPE Raman reporter [1,2-bis(4-pyridyl)ethylene] (BPE) and the aminosilane [aminopropyltrimethoxysilane (APTMS)] leads to the formation of a small population of aggregates. For the sake of brevity, we refer to these samples as SHINs. In comparison, the silica-capped aggregates in Figure 1B exhibit a high degree of anisotropy and polydispersity, with a greater number of AuNPs present within the aggregates. The DLS measurements in Figure 1C show an increase in size from 50 ± 1 to 63 ± 2 nm from the AuNP to the SHINs, due to the addition of the silica shell and some aggregation. The silica-capped aggregates have a size of 128 ± 6 nm from the combination of aggregation and their silica shell. These DLS values differ significantly from what is observed in the SEM images; it is important to note that DLS measures hydrodynamic radius and often has difficulty with highly anisotropic structures such as aggregates.^41^ Although NPs of >100 nm have difficulty entering cells, large NPs can still be used in photothermal therapy experiments. It has previously been shown that gold nanoshells with total diameters of ∼150 nm can be used for photothermal ablation of prostate tumors.^42^ In this case, the nanostructure accumulates within the tumor via the vasculature.

A comparison between the extinction spectra of the AuNPs, SHINs, and aggregates is shown in Figure 1D. For all characterization using extinction spectroscopy and SERS, samples were prepared to an optical density of 1 at a volume of 500 μL, based on the plasmon band present between 530 and 540 nm as this was the most consistent in terms of position and intensity between samples. In a previous study comparing nanoshells and “nanomatryoshkas”,^19^ the authors used an optical density of 1 to compare the different nanostructures; we therefore chose the same protocol. Since the LSPR is present in the AuNPs, we attribute the presence of this LSPR in both the SHINs and aggregates as originating from the AuNPs at their core. The slight shifts in the position of the LSPRs are subsequently attributed to the change in the refractive index at the surface of the nanostructures as the initial aqueous environment is replaced by silica. However, more interesting is the introduction of a new LSPR in both the SHINs and aggregates at 745 and 837 nm, respectively. This secondary NIR plasmon can be attributed to aggregation.^43^ The broadness of this plasmon is also related to the polydispersity of the aggregation, where differently sized shapes and aggregates are formed, and is notably less intense for the SHINs, suggesting a smaller population of aggregates, which is why this is not reflected in the DLS measurements but can be observed in the SEM images. This change in optical properties can also be observed visually, with AuNP appearing pink, SHINs appearing purple, and aggregates appearing indigo as shown in Figure S1.

Furthermore, the presence of this LSPR in the NIR region is of particular interest as NIR excitation wavelengths are beneficial for SERS measurements and photothermal measurements involving biological tissues. The induced aggregation additionally results in a strong SERS intensity in comparison to the SHINs as shown in Figure 1E. The increase can be attributed to the generation of nanoscale regions of strong electromagnetic field enhancement, referred to as hotspots, between the AuNPs within the aggregates. This is highly desirable as it suggests that the aggregates have the potential to be monitored at low concentrations if used in a biological application, such as photothermal therapy, where it is necessary to ensure that they are first in the correct location and that they have been expelled from the body in their entirety following treatment.

**Figure 2:**
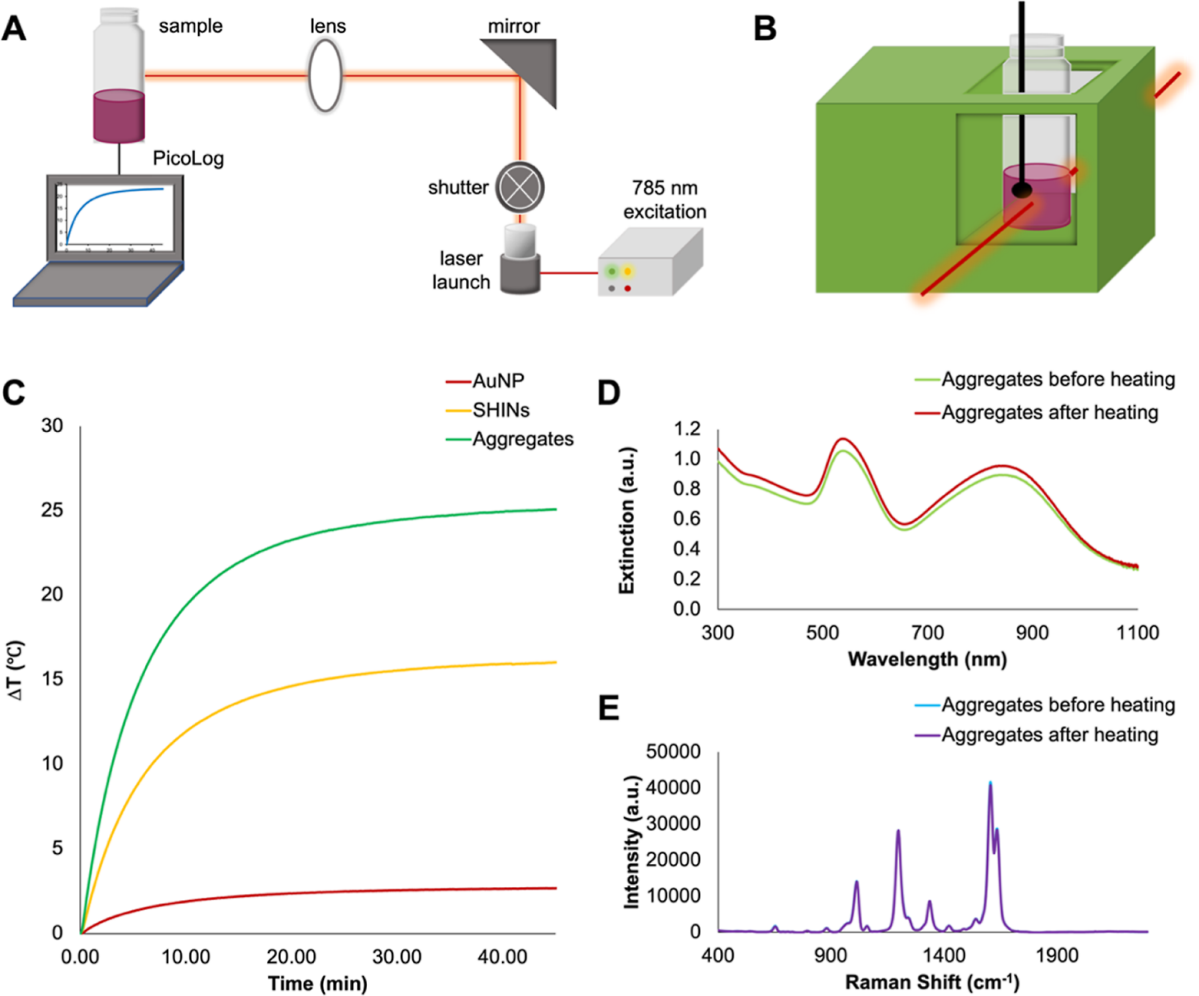
(A) Overview of in-house photothermal heating setup; the 785 nm laser beam is directed toward the sample using a mirror and lens. (B) Schematic representation of the sample in the cuvette holder in the path of the laser beam, with the temperature probe positioned so as not to interfere with the laser beam. (C) Change in temperature for AuNPs, SHINs, and aggregates when irradiated for 45 min at 785 nm, adjusted to an optical density of 1 based on their plasmon band at ∼540 nm. The temperature was digitally recorded at 1 s intervals from a starting temperature of 20.8 °C, using a thermocouple probe connected to PicoLog software. Comparison of (D) extinction, and (E) SERS spectra before and after heating the aggregates. Extinction spectra were collected using a Cary60 UV–vis spectrophotometer scanning from 300 to 1100 nm at a medium scanning rate of 600 nm/min. SERS spectra were collected using a hand-held Snowy Range Instruments CBEx spectrometer with an excitation wavelength of 785 nm, a laser power of 10 mW at the sample, and an acquisition time of 0.1 s. Following collection, spectra were baseline corrected using MATLAB (Version 2022b) and plotted in Excel. For characterization with extinction spectroscopy and SERS, samples were adjusted to an optical density of 1 for a volume of 500 μL as this was the sample volume used for heating experiments. All heating and characterization measurements were carried out in triplicate and averaged.

For heating experiments, a custom in-house heating setup shown in Figure 2A was used. Here, a 785 nm laser, with a maximum laser power of 300 mW at its output, was directed toward the sample using a series of mirrors and lenses. The colloidal nanoparticle solution (500 μL) was added into a glass vial (1.75 mL volume) and kept in place using a 3D printed holder. A thermocouple probe was placed within the solution to measure the temperature during heating experiments, positioned to not be within the path of the laser beam (Figure 2B), and the temperature was recorded at 1 s intervals using PicoLog software. Each sample was irradiated for 45 min to ensure that the equilibrium temperature was achieved.

The heating profiles for the various samples are shown in Figure 2C. After heating for 45 min, silica-capped AuNP aggregates outperformed the AuNPs and SHINs as shown by their heating profiles in Figure 2C where a temperature increase of 25.0 °C was achieved in comparison to only 2.7 and 16.0 °C for AuNP and SHINs, respectively. This supports the relationship between the NIR plasmon and the heating capability of the nanostructure. Before passing through the sample vial, the laser power was measured to be 279 mW, dropping to 235 mW after passing through the AuNPs, 147 mW after passing through SHINs, and 46 mW after passing through the aggregates. This confirms that more light is being absorbed by the SHINs and aggregates, contributing to the increased temperature. Furthermore, the increase in bulk temperature to 46 °C is especially promising as this is well within the range needed to cause hyperthermia-induced cell death.^14,15^

Following heating experiments, the nanostructures were characterized via extinction spectroscopy and SERS. Highlighted in Figure 2D,E are the silica-capped aggregates as these showed the greatest increase in temperature. The extinction spectra in Figure 2D show a slight increase in intensity after heating; we attribute this to evaporation of the solvent, concentrating the sample and therefore increasing the intensity. The plasmon band position remains unchanged at 538 nm, and the NIR plasmon at 837 nm experiences a small blue shift of 2 nm, now at 835 nm. This indicates the stability of the aggregates as the optical properties are largely unchanged after a high degree of heating for a prolonged period. Other types of nanostructures, such as nanorods, can undergo structural rearrangement following irradiation; in this instance, the anisotropic structures rearrange to form more thermodynamically stable spheres.^33^ Furthermore, as shown in Figure 2E, the SERS intensity is minimally affected after irradiation, with the most intense BPE peak decreasing from ∼42,000 counts to ∼39,000 counts, a change of approximately 7%. As these measurements were carried out at the lowest possible laser power and integration time (10 mW, 0.1 s) using a hand-held device, these small changes are negligible. We attribute these changes to solvent loss and interaction of nanostructures with the thermocouple probe during irradiation, which will slightly change the concentration of nanoparticles present and, therefore, can cause minor increases and decreases between samples. Overall, the minimal changes in the optical properties of silica-capped aggregates after such intense heating, for a prolonged period, indicate a high degree of stability.

Similar comparisons to the extinction and SERS spectra from before and after photothermal heating were also performed for the AuNPs and SHINs, and they are shown in Figure S2. Importantly, for both structures, the LSPR in the visible region remains unaffected. For the SHINs, the NIR LSPR undergoes a blue shift from 745 to 739 nm, but once again, this change is minor. There is also a small increase in extinction. We again attribute these differences before and after heating to solvent evaporation and nanostructures interacting with the probe during irradiation and affecting the concentration. Consistent with the aggregates, the SHINs experience a minimal decrease in their SERS intensity for the characteristic BPE peak following heating experiments, from ∼11,000 to ∼9000 counts. Given that silica-capped aggregates experience a temperature increase almost twice times that of the SHINs and ten times that of the AuNP, and exhibit an incredibly strong SERS intensity in comparison to the SHINs, the aggregates were selected for further investigation with regards to their heating capabilities.

### Heating Cycles

One method of evaluating the suitability of materials for photothermal therapy is to evaluate their stability after repeated cycles of heating and cooling. In the case of small molecules, such as fluorophores, it is often the case that after prolonged irradiation, the fluorophore is photobleached resulting in changes in optical properties and its heating efficiency.^44,45^ Since the aggregates experienced the largest increase in temperature, they were subjected to three 45 min heating cycles, again using a 785 nm laser excitation. By putting them through continuous cycles of heating and cooling, they can be further confirmed.

After each subsequent cycle, an increase in the bulk temperature was observed, increasing from 44.1 to 44.7 °C to 45.2 °C. We attribute the increase in temperature over time to some evaporation of the solution in the sample with each heating cycle as this will have effectively increased the concentration of nanostructures within the solution. Importantly, no decrease in temperature was observed as a decrease in temperature could imply significant changes to the optical and structural properties of the nanoparticles.

**Figure 3:**
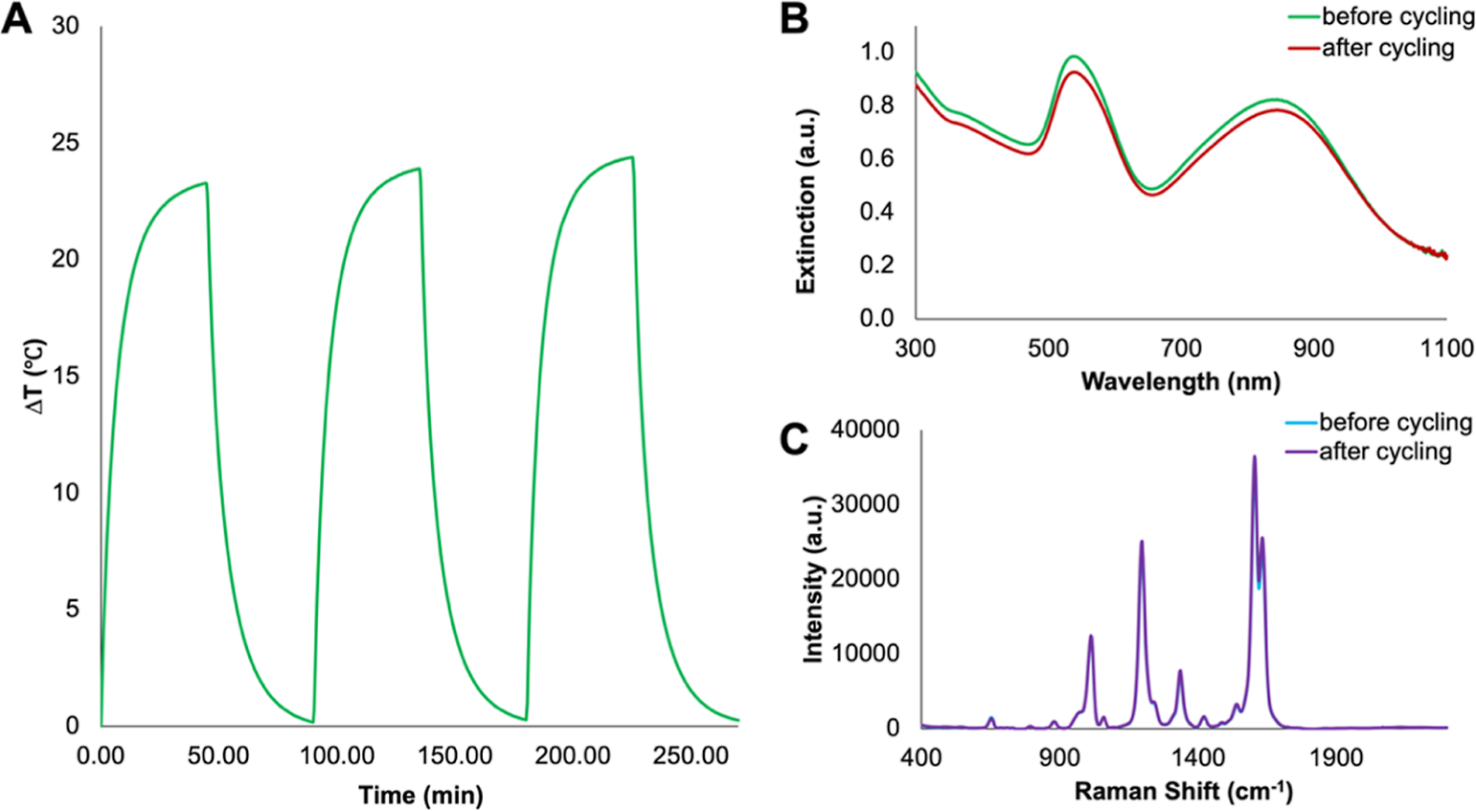
(A) Change in temperature (°C) for heating and cooling cycles for AuNP 500 nM BPE SiO_2_ aggregates at 45 min intervals, adjusted to an optical density of 1 based on the plasmon at ∼540 nm. The temperature was digitally recorded at 1 s intervals from a starting temperature of 20.8 °C, using a thermocouple probe connected to PicoLog software. (B) Extinction and (C) SERS spectra for AuNP 500 nM BPE SiO_2_ aggregates before and after cycling experiments. Extinction measurements were collected using a Cary60 UV–vis spectrophotometer scanning from 300 to 1100 nm at a medium scanning rate of 600 nm/min, and SERS spectra were collected using a hand-held Snowy Range Instruments CBEx spectrometer at an excitation wavelength of 785 nm, a laser power of 10 mW at the sample, and an acquisition time of 0.1 s. Following collection, spectra were baseline corrected using MATLAB (Version 2022b) and plotted in Excel. For characterization with extinction spectroscopy, and SERS samples were adjusted to an optical density of 1 for a volume of 500 μL.

Following three heating and cooling cycles, the aggregates were characterized using extinction spectroscopy (Figure 3B) and SERS (Figure 3C). Although the position of the first plasmon (538 nm) remains unchanged following heating cycles, there is a slight decrease in the intensity of this plasmon. Additionally, there is a red shift in the NIR plasmon from 837 to 844 nm; when normalized to an extinction of 1 for 450 nm, as shown in Figure S3, these changes are less apparent and importantly, the ratio of the extinction of the visible LSPR to the extinction of the NIR LSPR remain the same. The origin of this red shift is unclear, although it is possible that sample evaporation during repeated heating changes the populations of aggregates present, resulting in a higher population of larger aggregates which are absorbing at 844 nm instead of 837 nm. Although normalizing to 450 nm demonstrates smaller changes in the extinction, as the visible plasmon at ∼540 nm was used in determining optical density and preparing samples for heating, this is used for comparison of extinction before and after heating throughout the remainder of this work. There is an increase in the SERS intensity of the characteristic BPE peak at ∼1600 cm^–1^, from ∼35,000 counts to ∼36,000 counts; as before, this measurement was carried out at the lowest laser power and acquisition time the spectrometer allowed and so can be considered minimal. Overall, there are no changes to the extinction and SERS spectra that would indicate degradation of the aggregates, so it can be concluded that they remain stable and largely unchanged following repeated heating. Coupled with the high degree of structural and optical stability shown after repeated heating, the aggregates show promise as substrates for photothermal therapy, and key parameters in their synthesis can be further manipulated and investigated to improve their heating capabilities.

### Effect of the APTES Concentration on Optical Properties and Heating

During the synthesis of the aggregates, APTES contributes significantly to aggregation in addition to rendering the gold surface vitreophilic. Given that the position of the NIR LSPR is related to the aggregation of AuNPs, we chose to evaluate how the concentration of this aggregating agent changes the plasmonic and photothermal properties of the resulting nanostructures. To investigate the effects of the APTES concentration on aggregation and LSPR position, five batches of aggregates were synthesized with varying concentrations of APTES (6.5, 10.9, 16.2, 17.3, and 21.7 μM) with all other reagent concentrations being consistent with previously prepared aggregates. As with the previous study, the same batch of as-prepared AuNPs was used in synthesizing aggregates to ensure that any variations in properties were solely due to the concentration of APTES being varied and not due to variations in the AuNP concentration.

**Figure 4:**
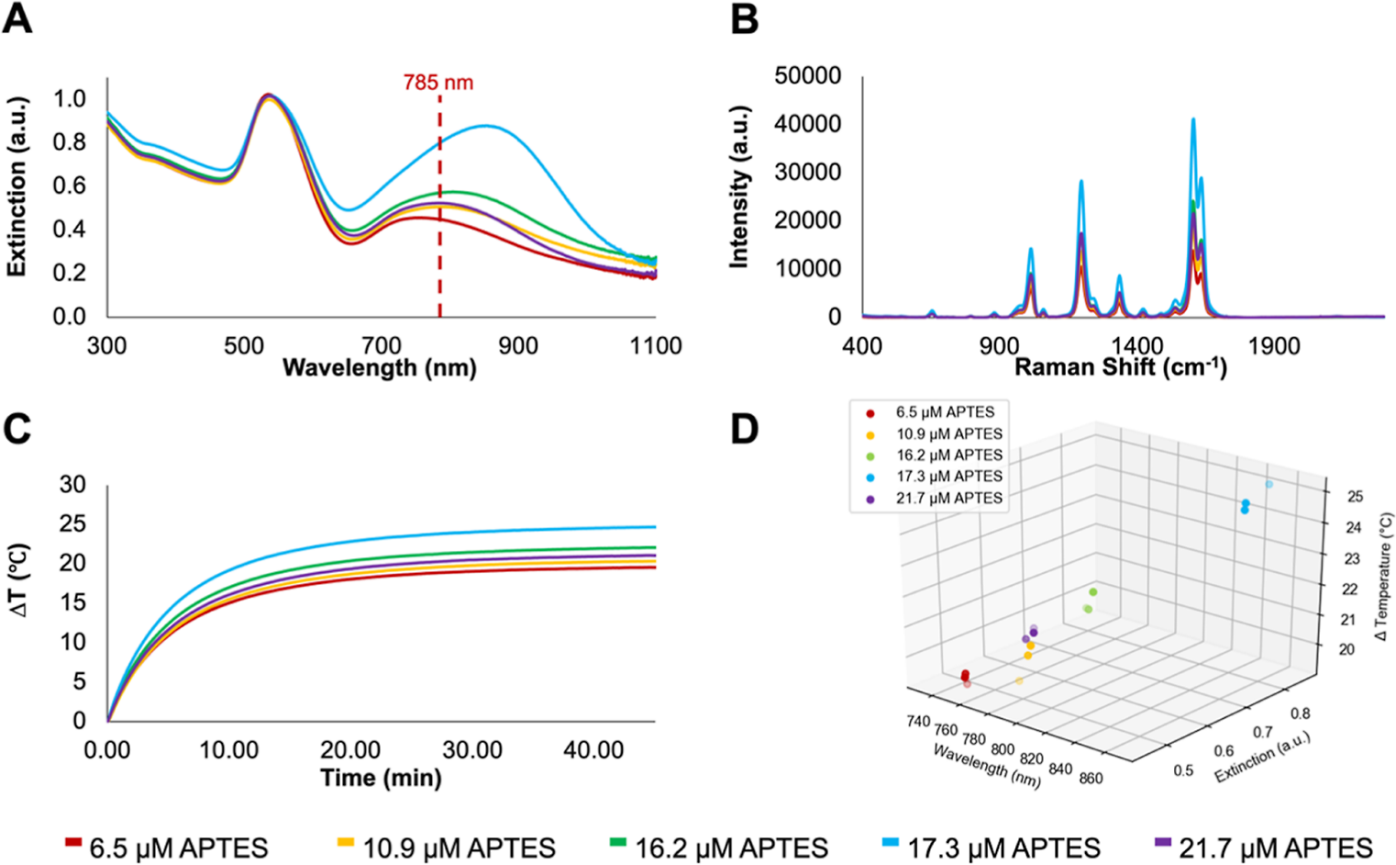
(A) Extinction spectra of AuNP BPE SiO_2_ aggregates with increasing APTES concentrations, collected using a Cary60 UV–vis spectrophotometer scanning from 300 to 1100 nm at a medium scanning rate of 600 nm/min. The excitation wavelength (785 nm) is indicated by a dashed line. (B) SERS spectra of aggregates with the increasing APTES concentration. SERS spectra were collected using a hand-held Snowy Range Instruments CBEx spectrometer at an excitation wavelength of 785 nm, a laser power of 10 mW at the sample, and an acquisition time of 0.1 s. Following collection, spectra were baseline corrected using MATLAB (Version 2022b) and plotted in Excel. (C) Change in temperature for the samples of aggregates when irradiated for 45 min at 785 nm, adjusted to an optical density of 1 based on their shared peak at ∼540 nm. The temperature was digitally recorded at 1 s intervals using a thermocouple probe connected to PicoLog software. (D) 3D scatter plot showing the change in temperature in relation to extinction and λ_max_ of the NIR plasmon.

As observed in the extinction spectra in Figure 4A, increasing the concentration of APTES leads to a general red shift and increase in intensity of the NIR LSPR, while the LSPR between 530 and 540 nm remained minimally affected. This indicates a general trend where increasing the APTES concentration leads to increasing the degree of aggregation of the AuNPs. There is also an increase in the hydrodynamic radius (Figure S4A) with the increasing APTES concentration. A notable exception to all of these trends is for the sample with the highest APTES concentration (21.7 μM). As opposed to further red-shifting, this sample blue-shifted relative to the previous concentrations (16.2 and 17.3 μM). We propose two mechanisms behind this blue shift. First, it is possible that there is a maximum degree of aggregation which can be achieved from an increased APTES concentration; not to mention, control over aggregation is inherently challenging. Second, larger aggregates may have settled out of solution, leading to a larger population of smaller aggregates within the irradiated regions, contributing to the observed blue-shifted plasmon; if this was the case, this was not visually apparent in the sample, pictured in Figure S4B. Unsurprisingly, the SERS intensity for the adsorbed BPE correlates with the plasmon position. The samples with the most red-shifted NIR plasmon (16.2 and 17.3 μM) had the strongest SERS intensity. As discussed earlier, this is due to increased aggregation increasing the number of hotspots present, therefore further enhancing the SERS signal.

The heating profiles for these samples are shown in Figure 4C. Consistent with the SERS results in Figure 4B, the most red-shifted samples had the greatest increase in temperature. To better correlate the optical properties with the observed increase in temperature, a 3D scatter plot was constructed that considers the position of the LSPR, the intensity of the extinction at that wavelength, and the increase in temperature (Figure 4D). The corresponding 2D representations are included in Figure S5 for clarity. Using these representations, it is possible to visualize the importance of both the LSPR position and the extinction intensity on the observed temperature increase. For example, although the 10.9 and 21.7 μM APTES samples have similar LSPR spectral positions, the 21.7 μM APTES sample has a slightly higher extinction and exhibits a greater increase in temperature. The dependence of temperature on both plasmon position and intensity offers an explanation as to why it is not necessarily those with NIR plasmons closest to the excitation wavelength that experience the greatest heating as one might expect. Instead, it is necessary to consider the population of aggregates within the sample, which is reflected in the extinction intensity. Although precise control over the effects of aggregation is out with the scope of this work, the ability to alter the optical properties, notably the position of the LSPR, does mean that the relationship between the LSPR and photothermal properties can be explored in greater detail.

Once again, a comparison was made between the various optical properties of the samples before and after heating, which are summarized in Figures S6, S7, and S8. The positions of both LSPRs remain within the standard deviations of each other (Figure S8A,C); the extinction values do change more after heating (Figure S8B,D), but importantly, the changes appear to be rather small. By considering the SERS intensity for the vibrational mode at ∼1605 cm^–1^ of the BPE spectra, only minimal changes in the SERS spectra were observed (Figure S8E). These results highlight the integrity of the aggregates after photothermal heating.

By a change in the APTES concentration, it becomes possible to adjust the state of aggregation of the nanoparticles and their optical properties. Both the position and intensity of this plasmon demonstrably contribute to the increase in temperature, and a λ_max_ closest to the excitation wavelength of 785 nm does not necessarily result in the highest temperature; indeed, it was those with further red-shifted and more intense NIR plasmons which exhibited the largest increase in temperature. Importantly, the optical properties remained stable after photothermal irradiation.

### Effect of Silica Shell Thickness on Optical Properties and Heating

Another important physical property of the silica-capped aggregates is the thickness of the silica shell, which influences the degree of aggregation during synthesis and enhances their stability. The thickness of the shell can be adjusted in two ways: (i) altering the concentration of silica added during initial shell growth and (ii) adding a second growth step using a modified Stöber process.^40^ In this process, TEOS undergoes hydrolysis and condensation in ethanol by means of an ammonium catalyst, further growing the silica shell on the nanoparticles.^46^ Details regarding the preparation of the samples with the thicker shell are in the Materials and Methods section. Here, we focus on the addition of TEOS and compare the optical properties and the increase in temperature during photothermal heating with and without the modified Stöber process for both SHINs and aggregates. To ensure that any optical and photothermal effects were solely due to the increased silica shell thickness, the same “parent” batches of SHINs and silica-capped aggregates from the initial comparison were used to prepare samples with an additional TEOS step and therefore thicker silica shell. This also ensured that the AuNP concentration remained the same for each sample. As was carried out throughout this work, the colloidal solutions were adjusted to an optical density of 1 for all characterizations.

**Figure 5:**
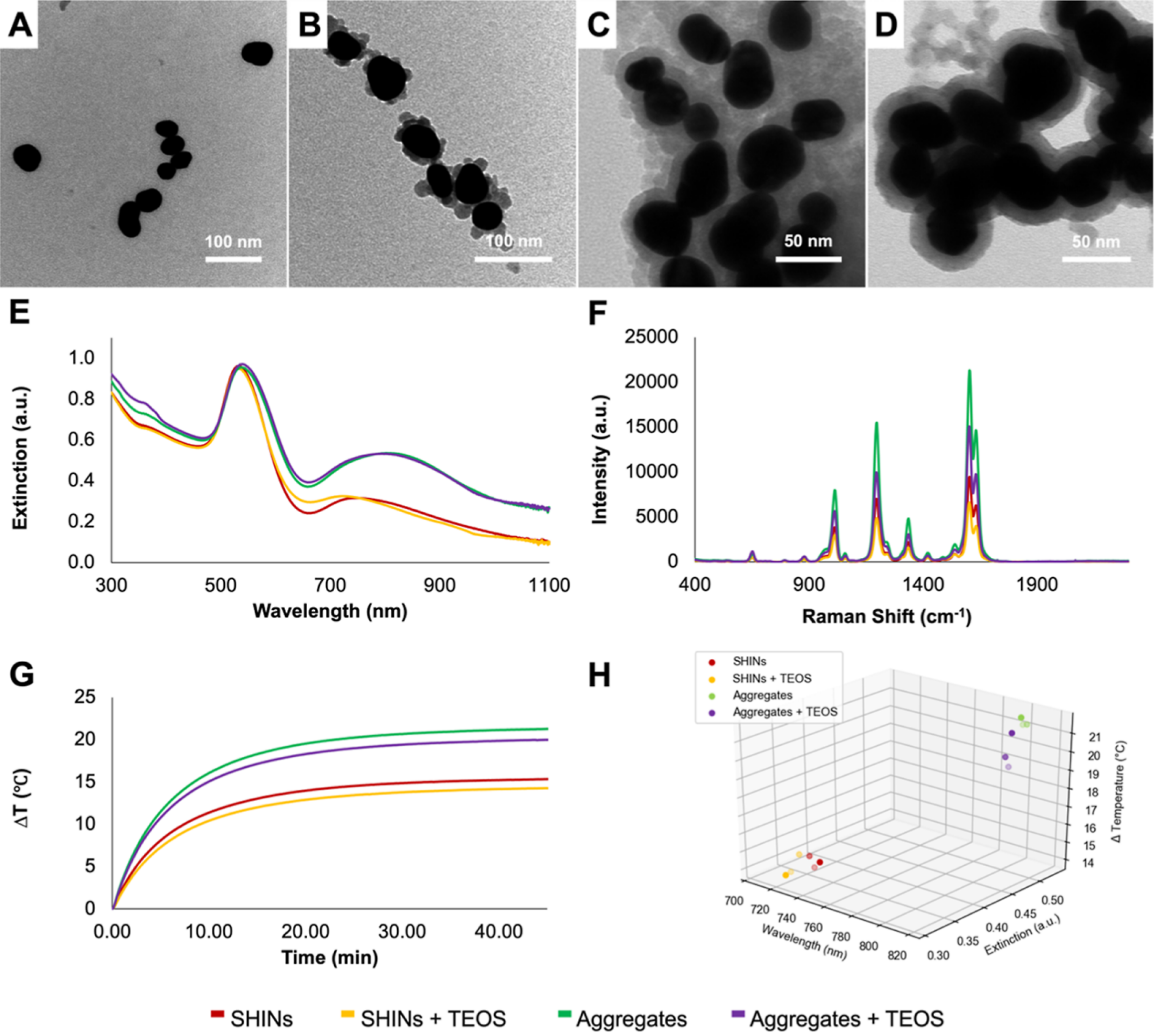
TEM images of SHINs prepared (A) without and (B) with TEOS, and silica-capped aggregates prepared (C) without and (D) with TEOS. (E) Extinction spectra of SHINs and aggregates prepared with and without TEOS, collected by using a Cary60 UV–vis spectrophotometer scanning from 300 to 1100 nm at a medium scanning rate of 600 nm/min. (F) SERS spectra of SHINs and aggregates prepared with and without TEOS. SERS spectra were collected using a hand-held Snowy Range Instruments CBEx spectrometer at an excitation wavelength of 785 nm, a laser power of 10 mW at the sample, and an acquisition time of 0.1 s. Following collection, spectra were baseline corrected using MATLAB (Version 2022b) and plotted in Excel. (G) Change in temperature for the samples of SHINs and aggregates when irradiated for 45 min at 785 nm, adjusted to an optical density of 1 based on their shared peak at ∼540 nm. The temperature was digitally recorded at 1 s intervals using a thermocouple probe connected to PicoLog software. (H) 3D scatter plot showing the change in temperature in relation to extinction and λ_max_ of the NIR plasmon.

To ensure that there was an increase in silica shell thickness following the addition of TEOS, samples were characterized by DLS and TEM. For both SHINs and aggregates, the DLS results showed an increase in size (Figure S9), indicating an increase in shell thickness. Given that DLS results are often less reliable for highly anisotropic structures, the shell thickness was also characterized by TEM images. These images are shown in Figure 5A–D, and additional TEM images can be found in Figure S10. To obtain a value for shell thickness, 25 measurements were taken of shell thickness at random points for each sample using ImageJ, and an average was calculated from these measurements. The shell thickness was confirmed to increase from 3.2 ± 1.0 to 6.3 ± 1.3 nm for SHINs, and 7.3 ± 0.9 to 10.7 ± 0.9 nm for aggregates. A full list of measurements can be found in Table S1. Interestingly, rather than a full shell being observed for SHINs following the addition of TEOS, it appears that discrete silica particles form during the Stöber process and instead “stack” onto the existing thin silica shell. This made it harder to accurately measure shell thickness due to the lack of a complete shell; however, importantly, there is a notable increase in the presence of silica surrounding the particles.

A comparison of the extinction spectra for the SHINs and aggregates with and without the additional TEOS step is shown in Figure 5E. Although the spectra for the aggregates appear very similar, the SHINs have a noticeable difference when the modified Stöber process is performed. The LSPR in the visible region remains similar, whereas the NIR plasmon resonance undergoes a slight blue shift from 749 to 722 nm. This is interesting as the addition of a silica shell to anisotropic nanostructures typically causes a red shift in the LSPR as the refractive index of silica is higher than that of water or air.^47−49^ Previous studies involving gold nanorods have observed blue shifts under certain conditions; blue shifts have been observed after purifying samples via centrifugation.^50,51^ In a different study, Mercadal et al. noticed that the porosity of the silica shell would influence the observed shift.^52^ In a porous shell, the LSPR had a blue shift, whereas a red shift was observed for nonporous silica shells. Though interesting, determining the porosity of the silica shell is outside the scope of this work. It is also known that for coupled nanoparticles, the position of the LSPR is sensitive to the interparticle spacing;^53^ this includes aggregates composed of silica-coated AuNPs.^54^ Once again, having various nanostructures with changes in the optical properties allows for more in-depth analyses into the relationship between LSPR position and extinction intensity.

Furthermore, as seen in Figure 5F, the addition of the TEOS step causes a noticeable decrease in the SERS intensity. The presence of the blue shift may be the cause, especially if it is due to slight changes in the interparticle spacing caused by the silica shell growing within these nanoscale gaps. This would result in a decrease in the electric field enhancement and ultimately a decrease in the SERS signal.^55^ Alternatively, some of the Raman reporter may be displaced during the growth of the thicker silica shell, which would also yield a weaker SERS response. Regardless, it was possible to easily observe the spectra at the lowest laser power of the hand-held Raman spectrometer used and an integration time of 0.1 s.

It was previously observed that increasing the thickness of a silica shell surrounding AuNPs did not have any appreciable effects on the observed heating.^29^ The results of Figure 5G indicate a similar general trend. Although there is a decrease in the observed temperature increase, the change is minimal (∼1 °C). To try to better understand this change, 3D scatter plots were once again prepared to consider any relationship between the LSPR position, intensity, and observed temperature increase (Figure 5H). 2D scatter plots for these parameters are shown in Figure S11. For the SHINs, samples prepared with and without TEOS have similar extinction values for the NIR LSPR, but the spectral positions are different. In this case, the sample of SHINs prepared with TEOS, with its blue-shifted NIR LSPR, had a lower temperature increase. The aggregates prepared with TEOS maintained the LSPR position but had a lower extinction. This also resulted in a lower temperature increase. This combination of contributing factors indicates that both parameters must be considered when trying to develop silica encapsulated nanostructures for photothermal applications.

To once again ensure that the optical properties were unaffected by photothermal heating, comparisons of the extinction and SERS spectra from before and after heating were made (Figures S12 and S13). Figure S14 provides a summary of those results. A blue shift of the visible LSPR position is observed for all samples after photothermal irradiation (Figure S14A,C); however, this change remains within the standard deviation for the three samples and is noticeably smaller for the two samples of aggregates in comparison to both samples of SHINs. As well, the extinction intensity is largely unchanged (Figure S14E). The NIR LSPR shows minimal change in both position and extinction, for all samples, after photothermal irradiation. Similarly, SERS intensity for the BPE peak at ∼1605 cm^–1^ in Figure S13E shows minimal change following heating (full SERS spectra are shown in Figure S13). As with all previous samples, this minimal change in optical properties demonstrates the strength of their structures and their suitability for photothermal heating. Additionally, it is promising that the addition of a thicker silica shell, which could improve long-term stability, does not result in a large decrease in temperature or dampening of SERS intensity.

## Conclusions

In summary, we have demonstrated the photothermal heating capabilities of AuNP-derived structures encapsulated with silica. By evaluating parameters crucial to their synthesis, it was possible to manipulate properties to investigate the relationship between the presence, position, and intensity of the NIR LSPR and the photothermal properties. Aggregate structures, formed by Raman reporter and aminosilane-induced aggregation of AuNPs that were then encapsulated in silica, outperformed both SHINs and as-prepared AuNPs under a 785 nm excitation. With bulk temperature increases nearing 25 °C, this illustrated the potential for this type of structure to be used in photothermal treatment. With this having been established, conditions related to the silica shell formation were explored for the preparation of the aggregates to better understand the relationship between the optical and photothermal properties. By adjusting the concentration of the secondary aggregating agent (APTES), it was found that there exists a relationship between the λ_max_ of the NIR plasmon and heating capability, whereby lowering the concentration of APTES and therefore degree of aggregation brought the NIR plasmon closer to the excitation wavelength of the laser used in heating and led to higher temperatures. Similarly, increasing the shell thickness through a modified Stöber process provided another means of slightly adjusting the optical properties of the aggregates. Once again, the importance of both the LSPR position and intensity plays a critical role in the observed temperature increase. The presence of the thicker shell had a greater impact on the SERS signal than on the measured bulk temperature. Throughout this work, comparisons were made between samples that were irradiated and samples that were not. Crucially, the positions and intensities of the LSPRs, and the SERS intensity, remained similar before and after heating. Overall, the ultrabright SERS properties can potentially enable tracking the distribution of the aggregates within a biological sample, such as a tumor, while the heat generated during photothermal irradiation subsequently kills the cells within the tumor. Since the optical properties are minimally affected by the photothermal irradiation, the aggregates can in principle then be traced again. As such, these aggregates are a promising new structure within the exciting fields of not only plasmonics but also cancer therapeutics.

## Data Availability

Research data associated with this work will become available through the following link: https://doi.org/10.15129/a0d6c841-9661-4967-b3f7-3c010583b412.

## Supporting Information Available

Materials characterization using DLS, zeta potential analysis, extinction spectroscopy, SERS spectra, and additional TEM images (PDF)

## References

[ref1] Wang Y.; Yan B.; Chen L. SERS Tags: Novel Optical Nanoprobes for Bioanalysis. Chem. Rev. 2013, 113 (3), 1391–1428. 10.1021/cr300120g. 23273312

[ref2] Jauffred L.; Samadi A.; Klingberg H.; Bendix P. M.; Oddershede L. B. Plasmonic Heating of Nanostructures. Chem. Rev. 2019, 119 (13), 8087–8130. 10.1021/acs.chemrev.8b00738. 31125213

[ref3] Khoshdel V.; Shokooh-Saremi M. Plasmonic nano bow-tie arrays with enhanced LSPR refractive index sensing. Micro Nano Lett. 2019, 14 (5), 566–571. 10.1049/mnl.2018.5588.

[ref4] Wallace G. Q.; Lagugné-Labarthet F. Advancements in fractal plasmonics: structures, optical properties, and applications. Analyst 2019, 144 (1), 13–30. 10.1039/C8AN01667D. 30403204

[ref5] Amendoeira A.; García L. R.; Fernandes A. R.; Baptista P. V. Light Irradiation of Gold Nanoparticles Toward Advanced Cancer Therapeutics. Adv. Ther. 2020, 3 (1), 1900153 10.1002/adtp.201900153.

[ref6] Willets K. A.; Van Duyne R. P. Localized Surface Plasmon Resonance Spectroscopy and Sensing. Annu. Rev. Phys. Chem. 2007, 58 (1), 267–297. 10.1146/annurev.physchem.58.032806.104607. 17067281

[ref7] Gellé A.; Jin T.; de la Garza L.; Price G. D.; Besteiro L. V.; Moores A. Applications of Plasmon-Enhanced Nanocatalysis to Organic Transformations. Chem. Rev. 2020, 120 (2), 986–1041. 10.1021/acs.chemrev.9b00187. 31725267

[ref8] Abadeer N. S.; Murphy C. J. Recent Progress in Cancer Thermal Therapy Using Gold Nanoparticles. J. Phys. Chem. C 2016, 120 (9), 4691–4716. 10.1021/acs.jpcc.5b11232.

[ref9] Tabish T. A.; Dey P.; Mosca S.; Salimi M.; Palombo F.; Matousek P.; Stone N. Smart Gold Nanostructures for Light Mediated Cancer Theranostics: Combining Optical Diagnostics with Photothermal Therapy. Adv. Sci. 2020, 7 (15), 1903441 10.1002/advs.201903441. PMC7404179 32775148

[ref10] Yang W.; Liang H.; Ma S.; Wang D.; Huang J. Gold nanoparticle based photothermal therapy: Development and application for effective cancer treatment. Sustain. Mater. Technol. 2019, 22, e00109 10.1016/j.susmat.2019.e00109.

[ref11] Bai X.; Wang Y.; Song Z.; Feng Y.; Chen Y.; Zhang D.; Feng L. The Basic Properties of Gold Nanoparticles and their Applications in Tumor Diagnosis and Treatment. Int. J. Mol. Sci. 2020, 21 (7), 2480 10.3390/ijms21072480. 32260051 PMC7178173

[ref12] Shan C.; Huang Y.; Wei J.; Chen M.; Wu L. Ultra-high thermally stable gold nanorods/radial mesoporous silica and their application in enhanced chemo-photothermal therapy. RSC Adv. 2021, 11 (18), 10416–10424. 10.1039/D1RA00213A. 35423593 PMC8695621

[ref13] Bian W.; Wang Y.; Pan Z.; Chen N.; Li X.; Wong W.-L.; Liu X.; He Y.; Zhang K.; Lu Y.-J. Review of Functionalized Nanomaterials for Photothermal Therapy of Cancers. ACS Appl. Nano Mater. 2021, 4 (11), 11353–11385. 10.1021/acsanm.1c01903.

[ref14] Zhang Y.; Zhan X.; Xiong J.; Peng S.; Huang W.; Joshi R.; Cai Y.; Liu Y.; Li R.; Yuan K.; et al. Temperature-dependent cell death patterns induced by functionalized gold nanoparticle photothermal therapy in melanoma cells. Sci. Rep. 2018, 8 (1), 8720 10.1038/s41598-018-26978-1. 29880902 PMC5992202

[ref15] Nam J.; Son S.; Park K. S.; Zou W.; Shea L. D.; Moon J. J. Cancer nanomedicine for combination cancer immunotherapy. Nat. Rev. Mater. 2019, 4 (6), 398–414. 10.1038/s41578-019-0108-1.

[ref16] Hirsch L. R.; Stafford R. J.; Bankson J. A.; Sershen S. R.; Rivera B.; Price R. E.; Hazle J. D.; Halas N. J.; West J. L. Nanoshell-mediated near-infrared thermal therapy of tumors under magnetic resonance guidance. Proc. Natl. Acad. Sci. U.S.A. 2003, 100 (23), 13549–13554. 10.1073/pnas.2232479100. 14597719 PMC263851

[ref17] Chatterjee H.; Rahman D. S.; Sengupta M.; Ghosh S. K. Gold Nanostars in Plasmonic Photothermal Therapy: The Role of Tip Heads in the Thermoplasmonic Landscape. J. Phys. Chem. C 2018, 122 (24), 13082–13094. 10.1021/acs.jpcc.8b00388.

[ref18] Huang X.; El-Sayed I. H.; Qian W.; El-Sayed M. A. Cancer Cell Imaging and Photothermal Therapy in the Near-Infrared Region by Using Gold Nanorods. J. Am. Chem. Soc. 2006, 128 (6), 2115–2120. 10.1021/ja057254a. 16464114

[ref19] Ayala-Orozco C.; Urban C.; Knight M. W.; Urban A. S.; Neumann O.; Bishnoi S. W.; Mukherjee S.; Goodman A. M.; Charron H.; Mitchell T.; et al. Au Nanomatryoshkas as Efficient Near-Infrared Photothermal Transducers for Cancer Treatment: Benchmarking against Nanoshells. ACS Nano 2014, 8 (6), 6372–6381. 10.1021/nn501871d. 24889266 PMC4076033

[ref20] Halas N. J.; Lal S.; Chang W.-S.; Link S.; Nordlander P. Plasmons in Strongly Coupled Metallic Nanostructures. Chem. Rev. 2011, 111 (6), 3913–3961. 10.1021/cr200061k. 21542636

[ref21] Wang Y.; Gao Z.; Han Z.; Liu Y.; Yang H.; Akkin T.; Hogan C. J.; Bischof J. C. Aggregation affects optical properties and photothermal heating of gold nanospheres. Sci. Rep. 2021, 11 (1), 898 10.1038/s41598-020-79393-w. 33441620 PMC7806971

[ref22] Sun M.; Liu F.; Zhu Y.; Wang W.; Hu J.; Liu J.; Dai Z.; Wang K.; Wei Y.; Bai J.; et al. Salt-induced aggregation of gold nanoparticles for photoacoustic imaging and photothermal therapy of cancer. Nanoscale 2016, 8 (8), 4452–4457. 10.1039/C6NR00056H. 26847879

[ref23] Pratap D.; Vikas; Gautam R.; Shaw A. K.; Soni S. Photothermal properties of stable aggregates of gold nanorods. Colloids Surf., A 2022, 635, 128054 10.1016/j.colsurfa.2021.128054.

[ref24] Roy S.; Kashyap R. K.; Pillai P. P. Thermoplasmonics Enable the Coupling of Light into the Solvent-Mediated Self-Assembly of Gold Nanoparticles. J. Phys. Chem. C 2023, 127 (21), 10355–10365. 10.1021/acs.jpcc.3c01316.

[ref25] Nam J.; Won N.; Jin H.; Chung H.; Kim S. pH-Induced Aggregation of Gold Nanoparticles for Photothermal Cancer Therapy. J. Am. Chem. Soc. 2009, 131 (38), 13639–13645. 10.1021/ja902062j. 19772360

[ref26] Kang S.; Bhang S. H.; Hwang S.; Yoon J.-K.; Song J.; Jang H.-K.; Kim S.; Kim B.-S. Mesenchymal Stem Cells Aggregate and Deliver Gold Nanoparticles to Tumors for Photothermal Therapy. ACS Nano 2015, 9 (10), 9678–9690. 10.1021/acsnano.5b02207. 26348606

[ref27] Park S.; Lee W. J.; Park S.; Choi D.; Kim S.; Park N. Reversibly pH-responsive gold nanoparticles and their applications for photothermal cancer therapy. Sci. Rep. 2019, 9 (1), 20180 10.1038/s41598-019-56754-8. 31882911 PMC6934723

[ref28] Yang S.; Yao D.; Wang Y.; Yang W.; Zhang B.; Wang D. Enzyme-triggered self-assembly of gold nanoparticles for enhanced retention effects and photothermal therapy of prostate cancer. Chem. Commun. 2018, 54 (70), 9841–9844. 10.1039/C8CC05136D. 30110025

[ref29] Penelas M. J.; Arenas G. F.; Trabadelo F.; Soler-Illia G.; Moya S. E.; Angelomé P. C.; Hoppe C. E. Importance of the Structural and Physicochemical Properties of Silica Nanoshells in the Photothermal Effect of Silica-Coated Au Nanoparticles Suspensions. Langmuir 2022, 38 (12), 3876–3886. 10.1021/acs.langmuir.2c00127. 35302776

[ref30] Sztandera K.; Gorzkiewicz M.; Klajnert-Maculewicz B. Gold Nanoparticles in Cancer Treatment. Mol. Pharmaceutics 2019, 16 (1), 1–23. 10.1021/acs.molpharmaceut.8b00810. 30452861

[ref31] Sloan-Dennison S.; Bevins M. R.; Scarpitti B. T.; Sauvé V. K.; Schultz Z. D. Protein corona-resistant SERS tags for live cell detection of integrin receptors. Analyst 2019, 144 (18), 5538–5546. 10.1039/C9AN01056D. 31402356 PMC6733675

[ref32] Sloan-Dennison S.; Laing S.; Graham D.; Faulds K. From Raman to SESORRS: moving deeper into cancer detection and treatment monitoring. Chem. Commun. 2021, 57 (93), 12436–12451. 10.1039/D1CC04805H. PMC8609625 34734952

[ref33] Taylor A. B.; Siddiquee A. M.; Chon J. W. M. Below Melting Point Photothermal Reshaping of Single Gold Nanorods Driven by Surface Diffusion. ACS Nano 2014, 8 (12), 12071–12079. 10.1021/nn5055283. 25405517

[ref34] Zhang W.; Jiang L.; Piper J. A.; Wang Y. SERS Nanotags and Their Applications in Biosensing and Bioimaging. J. Anal. Test. 2018, 2 (1), 26–44. 10.1007/s41664-018-0053-9.

[ref35] Yin B.; Ho W. K. H.; Xia X.; Chan C. K. W.; Zhang Q.; Ng Y. M.; Lam C. Y. K.; Cheung J. C. W.; Wang J.; Yang M.; et al. A Multilayered Mesoporous Gold Nanoarchitecture for Ultraeffective Near-Infrared Light-Controlled Chemo/Photothermal Therapy for Cancer Guided by SERS Imaging. Small 2023, 19, 2206762 10.1002/smll.202206762. 36593512

[ref36] Berry M. E.; McCabe S. M.; Sloan-Dennison S.; Laing S.; Shand N. C.; Graham D.; Faulds K. Tomographic Imaging and Localization of Nanoparticles in Tissue Using Surface-Enhanced Spatially Offset Raman Spectroscopy. ACS Appl. Mater. Interfaces 2022, 14 (28), 31613–31624. 10.1021/acsami.2c05611. 35801671 PMC9305698

[ref37] Turkevich J.; Stevenson P. C.; Hillier J. A study of the nucleation and growth processes in the synthesis of colloidal gold. Discuss. Faraday Soc. 1951, 11 (0), 55–75. 10.1039/df9511100055.

[ref38] Li J. F.; Huang Y. F.; Ding Y.; Yang Z. L.; Li S. B.; Zhou X. S.; Fan F. R.; Zhang W.; Zhou Z. Y.; Wu D. Y.; et al. Shell-isolated nanoparticle-enhanced Raman spectroscopy. Nature 2010, 464 (7287), 392–395. 10.1038/nature08907. 20237566

[ref39] Li J. F.; Tian X. D.; Li S. B.; Anema J. R.; Yang Z. L.; Ding Y.; Wu Y. F.; Zeng Y. M.; Chen Q. Z.; Ren B.; et al. Surface analysis using shell-isolated nanoparticle-enhanced Raman spectroscopy. Nat. Protoc. 2013, 8 (1), 52–65. 10.1038/nprot.2012.141. 23237829

[ref40] Stöber W.; Fink A.; Bohn E. Controlled growth of monodisperse silica spheres in the micron size range. J. Colloid Interface Sci. 1968, 26 (1), 62–69. 10.1016/0021-9797(68)90272-5.

[ref41] Zheng T.; Bott S.; Huo Q. Techniques for Accurate Sizing of Gold Nanoparticles Using Dynamic Light Scattering with Particular Application to Chemical and Biological Sensing Based on Aggregate Formation. ACS Appl. Mater. Interfaces 2016, 8 (33), 21585–21594. 10.1021/acsami.6b06903. 27472008

[ref42] Rastinehad A. R.; Anastos H.; Wajswol E.; Winoker J. S.; Sfakianos J. P.; Doppalapudi S. K.; Carrick M. R.; Knauer C. J.; Taouli B.; Lewis S. C.; et al. Gold nanoshell-localized photothermal ablation of prostate tumors in a clinical pilot device study. Proc. Natl. Acad. Sci. U.S.A. 2019, 116 (37), 18590–18596. 10.1073/pnas.1906929116. 31451630 PMC6744844

[ref43] Kearns H.; Bedics M. A.; Shand N. C.; Faulds K.; Detty M. R.; Graham D. Sensitive SERS nanotags for use with 1550 nm (retina-safe) laser excitation. Analyst 2016, 141 (17), 5062–5065. 10.1039/C5AN02662H. 26788554

[ref44] Huang X.; Jain P. K.; El-Sayed I. H.; El-Sayed M. A. Plasmonic photothermal therapy (PPTT) using gold nanoparticles. Lasers Med. Sci. 2008, 23 (3), 217–228. 10.1007/s10103-007-0470-x. 17674122

[ref45] Yuan A.; Wu J.; Tang X.; Zhao L.; Xu F.; Hu Y. Application of Near-Infrared Dyes for Tumor Imaging, Photothermal, and Photodynamic Therapies. J. Pharm. Sci. 2013, 102 (1), 6–28. 10.1002/jps.23356. 23132644

[ref46] Han Y.; Lu Z.; Teng Z.; Liang J.; Guo Z.; Wang D.; Han M.-Y.; Yang W. Unraveling the Growth Mechanism of Silica Particles in the Stöber Method: In Situ Seeded Growth Model. Langmuir 2017, 33 (23), 5879–5890. 10.1021/acs.langmuir.7b01140. 28514596

[ref47] Yasukuni R.; Ouhenia-Ouadahi K.; Boubekeur-Lecaque L.; Félidj N.; Maurel F.; Métivier R.; Nakatani K.; Aubard J.; Grand J. Silica-Coated Gold Nanorod Arrays for Nanoplasmonics Devices. Langmuir 2013, 29 (41), 12633–12637. 10.1021/la402810e. 24070218

[ref48] Wu W.-C.; Tracy J. B. Large-Scale Silica Overcoating of Gold Nanorods with Tunable Shell Thicknesses. Chem. Mater. 2015, 27 (8), 2888–2894. 10.1021/cm504764v. 26146454 PMC4486371

[ref49] Wang M.; Hoff A.; Doebler J. E.; Emory S. R.; Bao Y. Dumbbell-Like Silica Coated Gold Nanorods and Their Plasmonic Properties. Langmuir 2019, 35 (51), 16886–16892. 10.1021/acs.langmuir.9b03133. 31710809

[ref50] Pellas V.; Blanchard J.; Guibert C.; Krafft J.-M.; Miche A.; Salmain M.; Boujday S. Gold Nanorod Coating with Silica Shells Having Controlled Thickness and Oriented Porosity: Tailoring the Shells for Biosensing. ACS Appl. Nano Mater. 2021, 4 (9), 9842–9854. 10.1021/acsanm.1c02297.

[ref51] Meyer S. M.; Murphy C. J. Anisotropic silica coating on gold nanorods boosts their potential as SERS sensors. Nanoscale 2022, 14 (13), 5214–5226. 10.1039/D1NR07918B. 35315863

[ref52] Mercadal P. A.; Perez L. A.; Coronado E. A. Optical Properties of Silica-Coated Au Nanorods: Correlating Theory and Experiments for Determining the Shell Porosity. J. Phys. Chem. C 2021, 125 (28), 15516–15526. 10.1021/acs.jpcc.1c02647.

[ref53] Hill R. T.; Mock J. J.; Hucknall A.; Wolter S. D.; Jokerst N. M.; Smith D. R.; Chilkoti A. Plasmon Ruler with Angstrom Length Resolution. ACS Nano 2012, 6 (10), 9237–9246. 10.1021/nn3035809. 22966857 PMC3525076

[ref54] Vanderkooy A.; Chen Y.; Gonzaga F.; Brook M. A. Silica Shell/Gold Core Nanoparticles: Correlating Shell Thickness with the Plasmonic Red Shift upon Aggregation. ACS Appl. Mater. Interfaces 2011, 3 (10), 3942–3947. 10.1021/am200825f. 21882833

[ref55] Hao E.; Schatz G. C. Electromagnetic fields around silver nanoparticles and dimers. J. Chem. Phys. 2004, 120 (1), 357–366. 10.1063/1.1629280. 15267296

