# Supplementary material for: Plasmonic and Photothermal Properties of Silica-Capped Gold Nanoparticle Aggregates
Source: J Phys Chem C Nanomater Interfaces. 2023 Dec 12;127(50):24475–86. doi: 10.1021/acs.jpcc.3c07536 (PMC10749475; doi:10.1021/acs.jpcc.3c07536)
Supplement: Supplementary file 1 — jp3c07536_si_001.pdf [file jp3c07536_si_001.pdf]

Plasmonic and Photothermal Properties of Silica-Capped Gold Nanoparticle Aggregates

Jodie Fergusson,[1] Gregory Q. Wallace,[1] Sian Sloan-Dennison,[1] Ruairí Carland,[1] Neil C. Shand,[2] Duncan Graham,[1] and Karen Faulds[1]*

[1]Centre for Nanometrology, Department of Pure and Applied Chemistry, Technology and Innovation Centre, 90 George Street, Glasgow, G1 1RD
[2]Defence Science and Technology Laboratory, Porton Down, Salisbury, SP4 0JQ

SUPPLEMENTARY INFORMATION

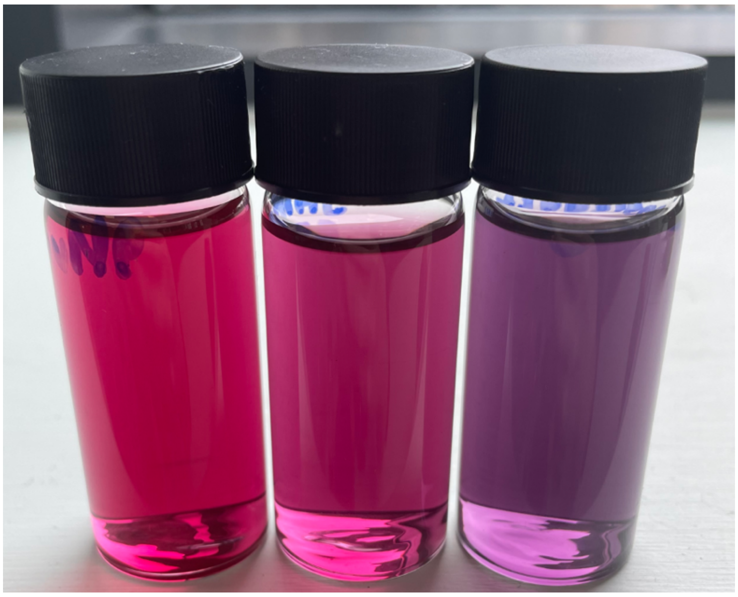

Figure S1: From left to right: as-prepared gold nanoparticles, SHINs, and silica-capped aggregates.

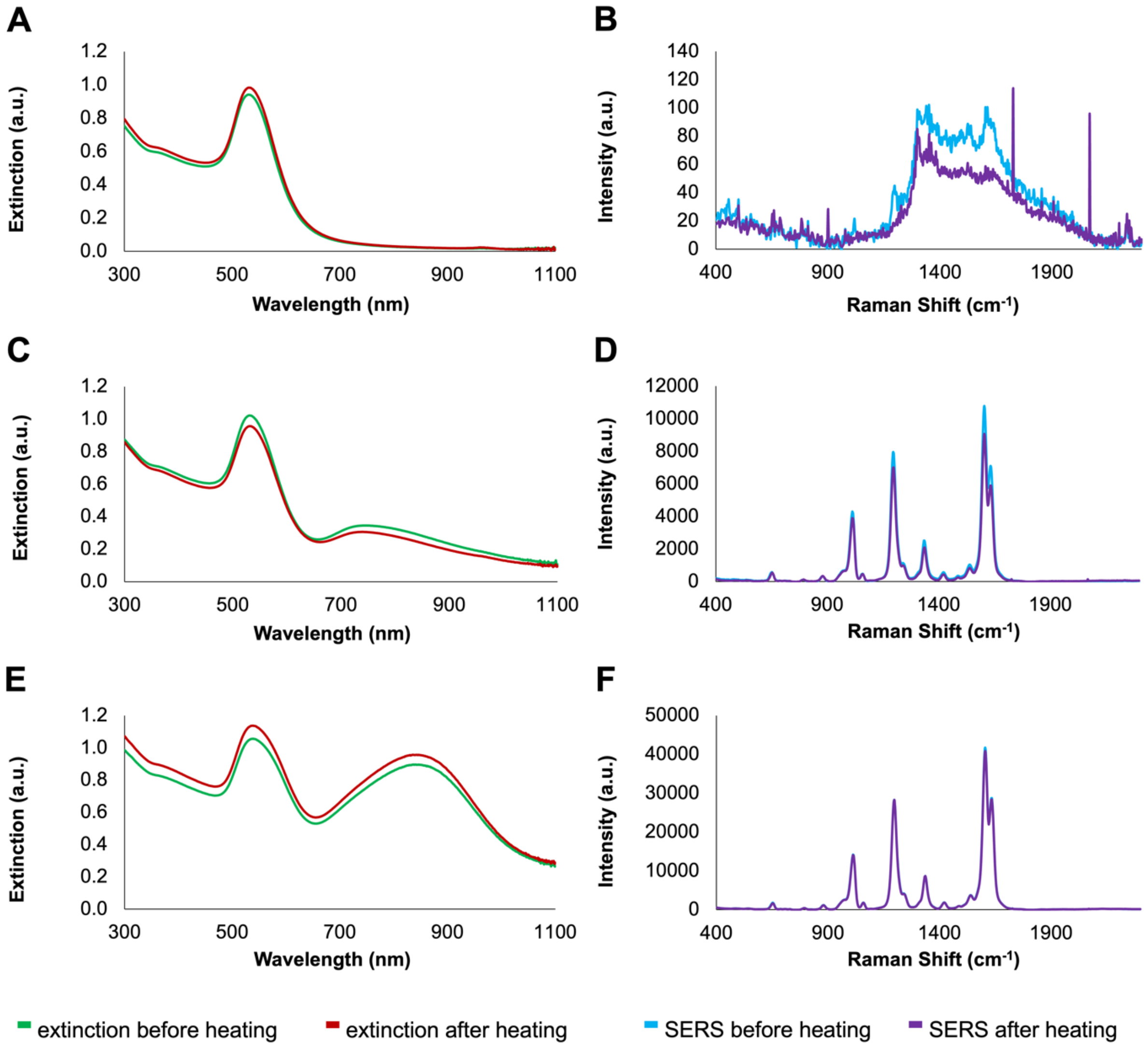

Figure S2: Extinction spectra before and after photothermal heating of (A) as-prepared gold nanoparticles, (B) SHINs, and (C) silica-capped aggregates, collected using a Cary-60 UV-vis spectrometer scanning from 300 nm to 1100 nm at a scanning rate of 600 nm/min. SERS spectra before and after photothermal heating of (D) as-prepared gold nanoparticles, (E) SHINs, and (F) silica-capped aggregates, collected using a Snowy Range Instruments CBex handheld spectrometer at an excitation wavelength of 785 nm, laser power of 10 mW, and acquisition time 0.1 s. SERS spectra were baseline corrected using MatLab (Version 2022b) and plotted in Excel. Samples were adjusted to an optical density of 1.

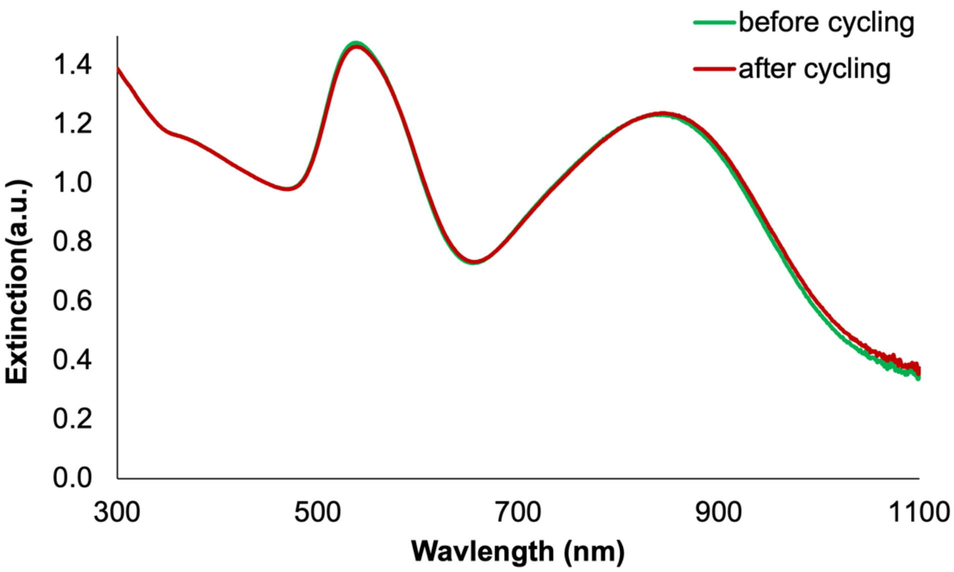

Figure S3: Extinction spectrum of silica-capped aggregates before and after cycling experiments, normalised to 1 for 450 nm, collected using a Cary-60 UV-vis spectrometer scanning from 300 nm to 1100 nm at a scanning rate of 600 nm/min.

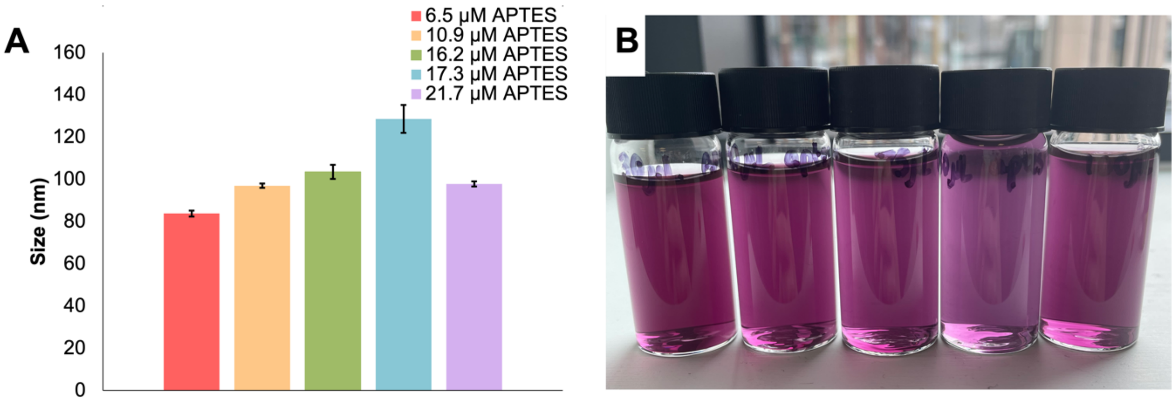

Figure S4: (A) Dynamic light scattering measurements of the hydrodynamic radii for silica-capped aggregates with increasing concentrations of APTES, collected using a Malvern Nanoseries Zetasizer. Samples were analysed as-prepared. (B) Silica-capped aggregates prepared with (L-R): 6.5, 10.9, 16.2, 17.3, and 21.7 µM APTES.

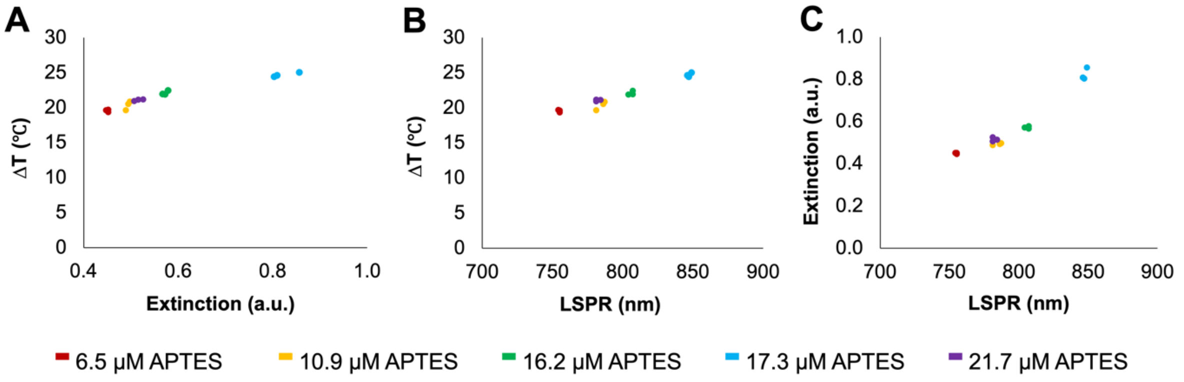

Figure S5: Scatter plots showing relationship between (A) extinction and change in temperature, (B) LSPR and change in temperature, and (C) LSPR and extinction, for five samples of silica-capped aggregates with increasing concentrations of APTES.

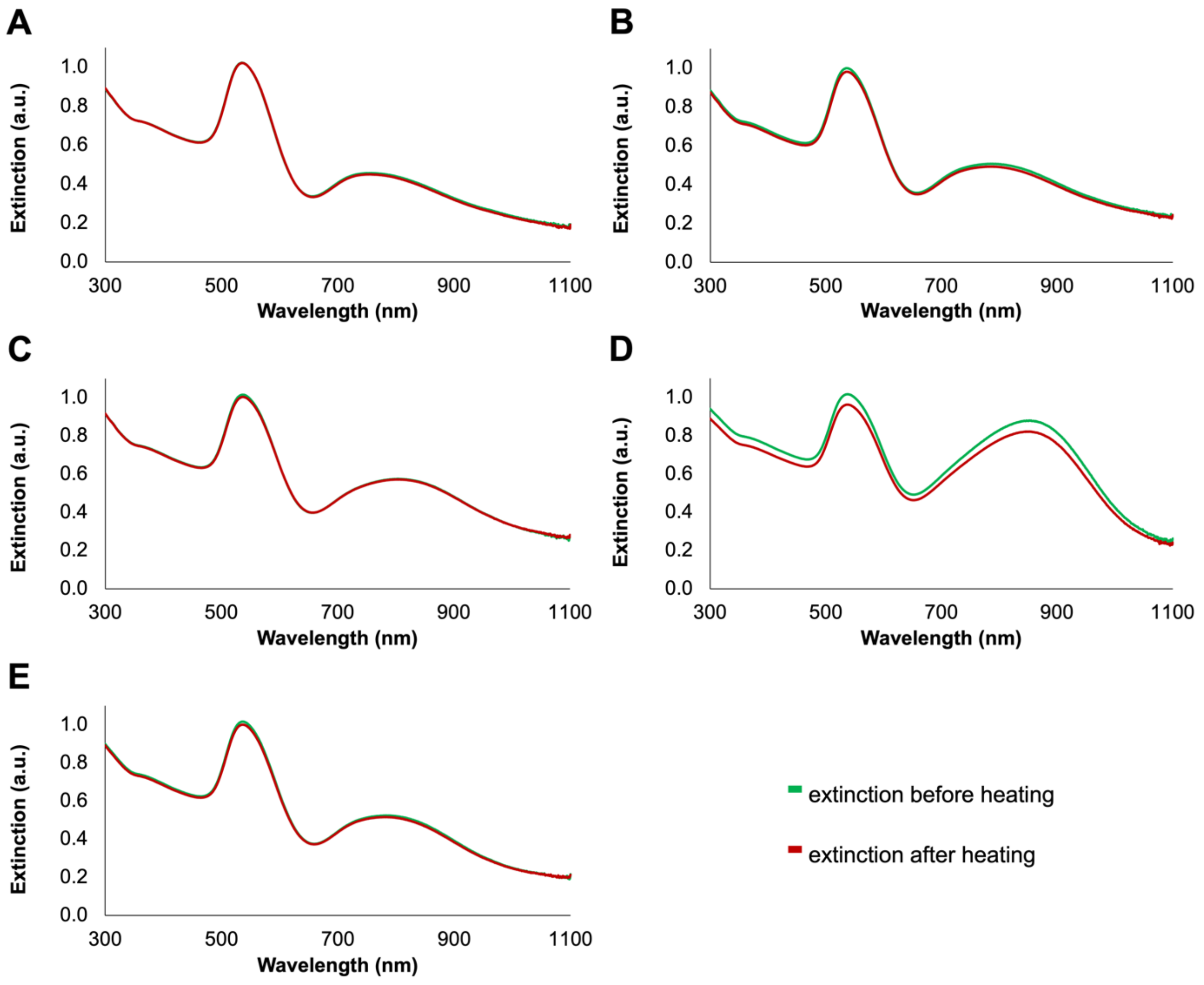

Figure S6: Extinction spectra before and after photothermal heating for silica-capped aggregates with (A) 6.5 µM, (B) 10.9 µM, (C) 16.2 µM, (D) 17.3 µM, and (E) 21.7 µM APTES, collected using a Cary-60 UV-vis spectrometer scanning from 300 nm to 1100 nm at a scanning rate of 600 nm/min. Samples were adjusted to an optical density of 1 for a volume of 500 µL.

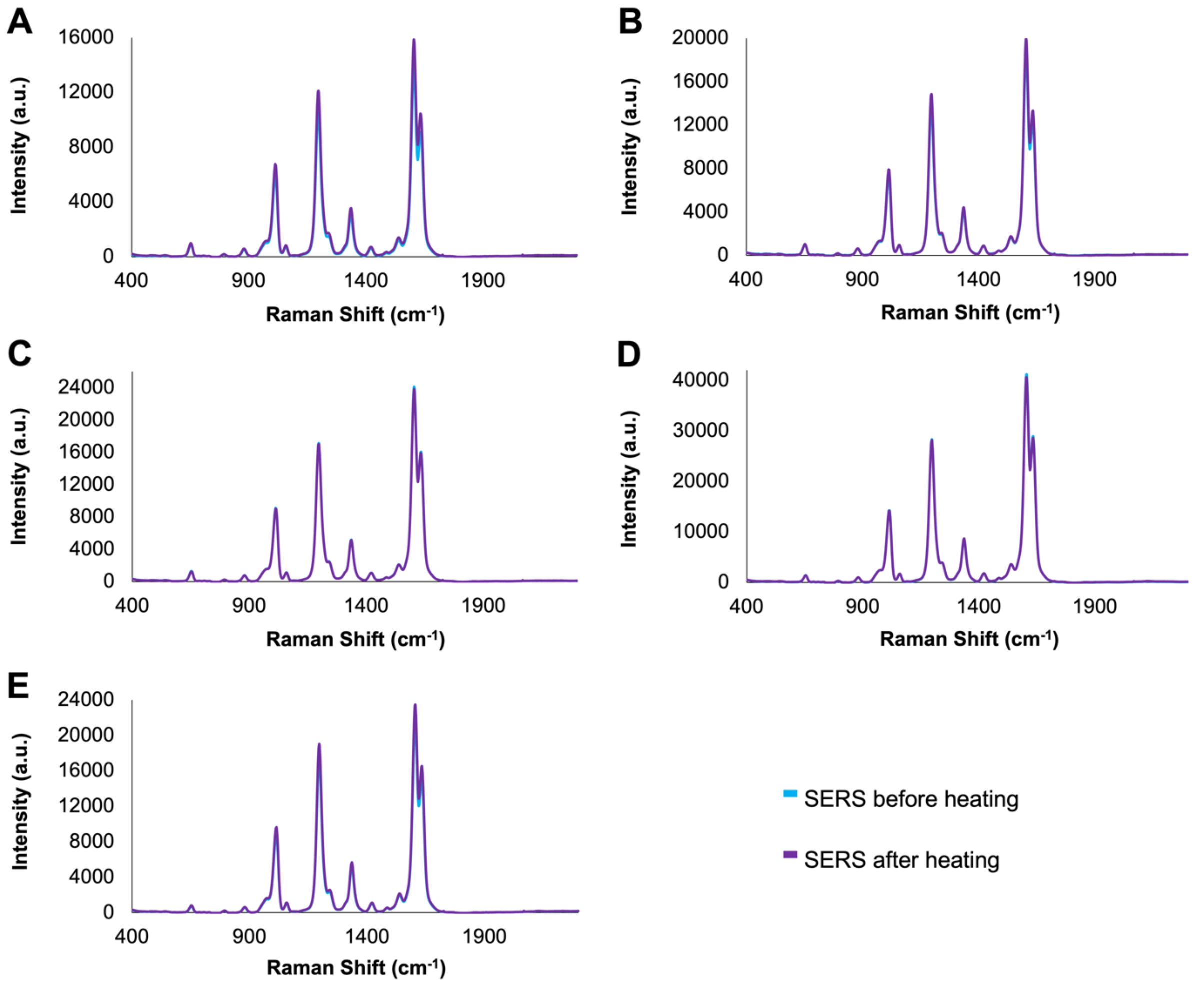

Figure S7: SERS spectra before and after photothermal heating for silica-capped aggregates with (A) 6.5 µM, (B) 10.9 µM, (C) 16.2 µM, (D) 17.3 µM, and (E) 21.7 µM APTES, collected using a Snowy Range Instruments CBex handheld spectrometer at an excitation wavelength of 785 nm, laser power of 10 mW, and acquisition time 0.1 s. SERS spectra were baseline corrected using MatLab (Version 2022b) and plotted in Excel. Samples were adjusted to an optical density of 1 for a volume of 500 µL.

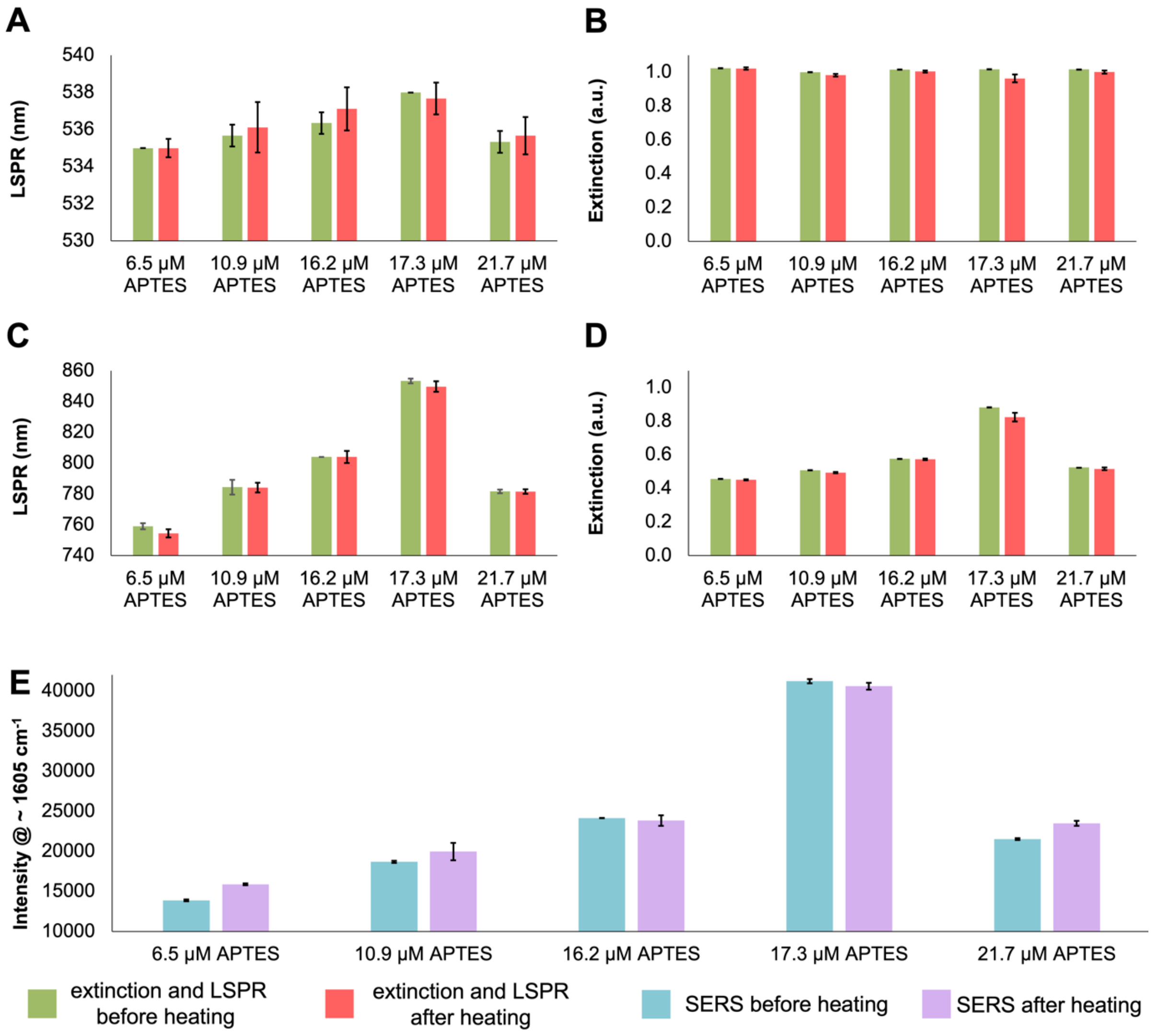

Figure S8. Changes in the (A) position and (B) extinction intensity of the LSPR in the visible region, and (C) position and (D) extinction intensity of the NIR LSPR for aggregates prepared using different concentrations of APTES before and after photothermal heating. Extinction spectra were collected using a Cary60 UV-vis spectrophotometer scanning from 300 to 1100 nm at a medium scanning rate of 600 nm/min. (E) Changes in the BPE SERS peak intensity at ~ 1605 cm^{-1} for the same types of aggregates before and after heating. SERS spectra were collected using a handheld Snowy Range Instruments CBex spectrometer at an excitation wavelength of 785 nm, laser power of 10 mW at the sample, and acquisition time of 0.1 s. Following collection, spectra were baseline corrected using MatLab (Version 2022b) and plotted in Excel. For characterisation with extinction spectroscopy and SERS, samples were adjusted to an optical density of 1 for a volume of 500 μL.

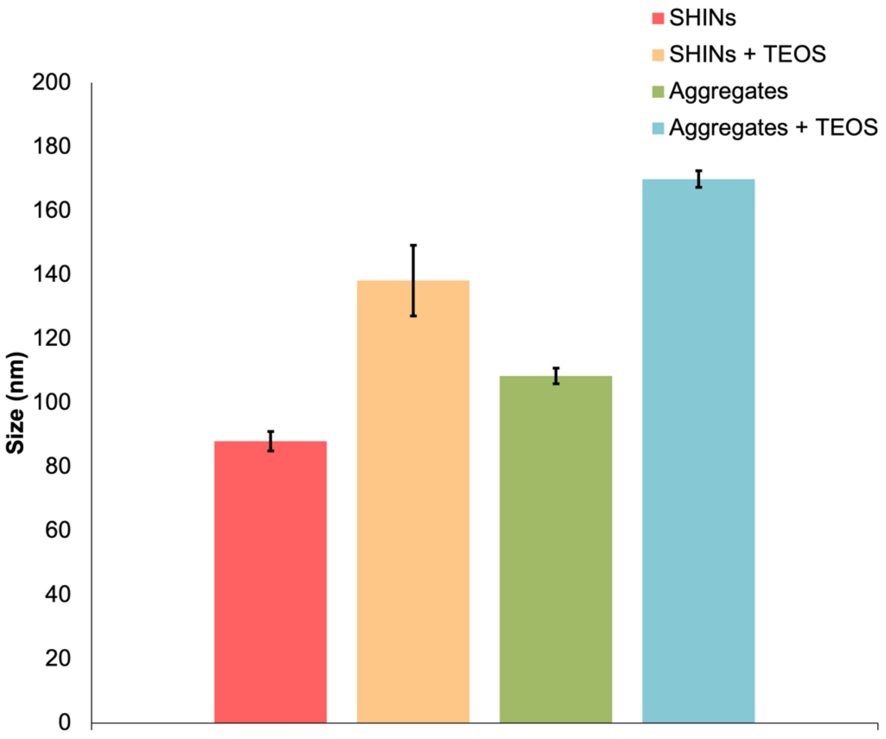

Figure S9: Dynamic light scattering measurements of the hydrodynamic radii for SHINs and aggregates prepared with and without an additional TEOS shell, collected using a Malvern Nanoseries Zetasizer. Samples were analysed as-prepared.

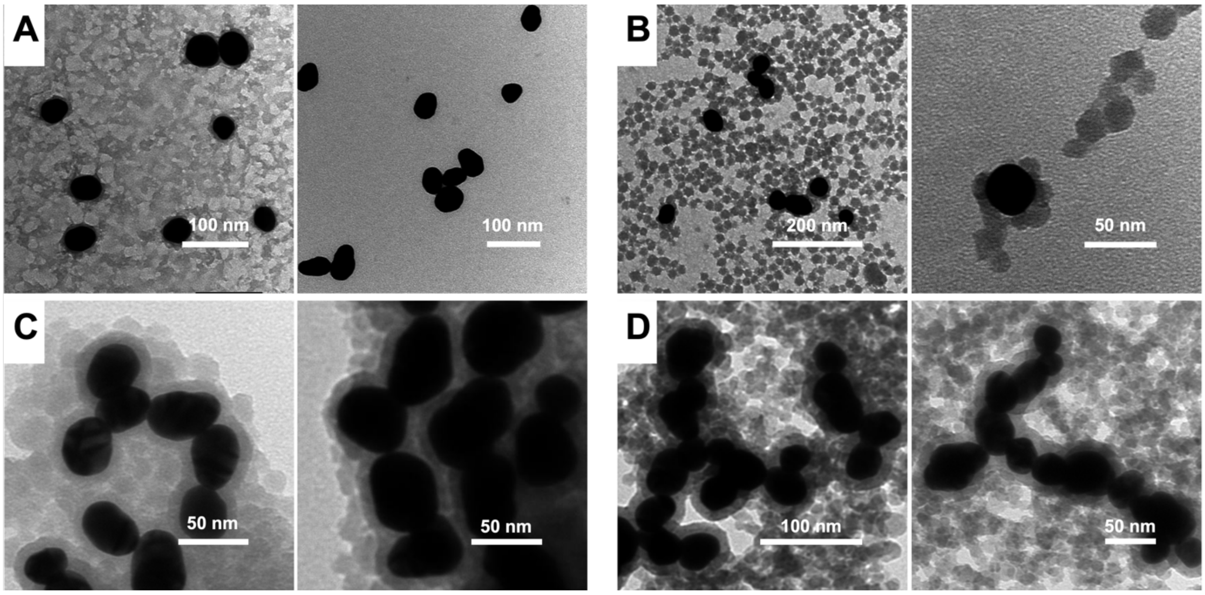

Figure S10: Additional TEM images of SHINs prepared (A) without and (B) with TEOS; silica-capped aggregates prepared (C) without and (D) with TEOS.

Table S1: Silica shell thickness measurements (nm) for SHINs, SHINs + TEOS, aggregates, and aggregates prepared with TEOS. Sizes were obtained using ImageJ and measuring 25 random points of the shell for each type of sample.

Measurement	Silica Shell Thickness (nm)			
	SHINs	SHINs + TEOS	Aggregates	Aggregates + TEOS
1	1.845	5.826	8.741	11.267
2	2.610	7.076	8.846	12.306
3	2.323	5.469	7.668	11.563
4	2.594	5.249	8.450	11.867
5	2.939	5.797	6.643	11.453
6	3.063	6.591	7.676	10.111
7	1.845	4.297	8.230	10.781
8	3.115	5.499	8.640	10.862
9	3.888	5.594	7.806	12.596
10	2.415	8.022	6.481	9.033
11	1.833	8.451	6.257	9.828
12	2.255	4.084	6.357	9.710
13	3.764	7.891	7.826	10.090
14	4.278	4.877	6.981	10.093
15	3.508	6.559	6.031	10.021
16	4.915	8.059	7.333	10.769
17	3.017	5.598	7.170	10.725
18	4.514	5.321	6.875	10.312
19	2.894	7.264	6.970	11.796
20	2.761	8.361	6.102	10.636
21	4.612	6.673	7.011	9.426
22	5.247	5.575	7.741	10.632
23	2.169	6.481	6.522	10.361
24	3.395	5.415	6.970	10.156
25	3.128	7.917	6.419	11.269
**Average**	3.157	6.318	7.270	10.707
**Standard Deviation**	0.977	1.276	0.855	0.890

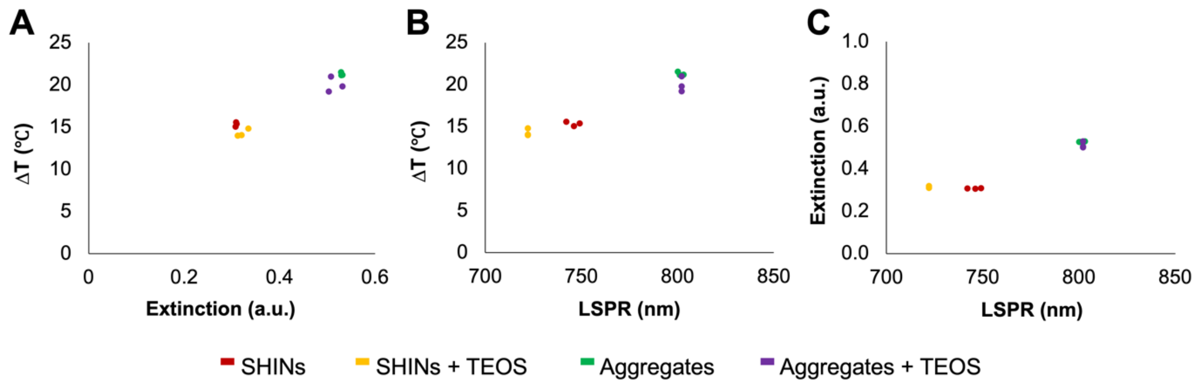

Figure S11: Scatter plots showing relationship between (A) extinction and change in temperature, (B) LSPR and change in temperature, and (C) LSPR and extinction, for SHINs and aggregates prepared with and without an additional TEOS shell.

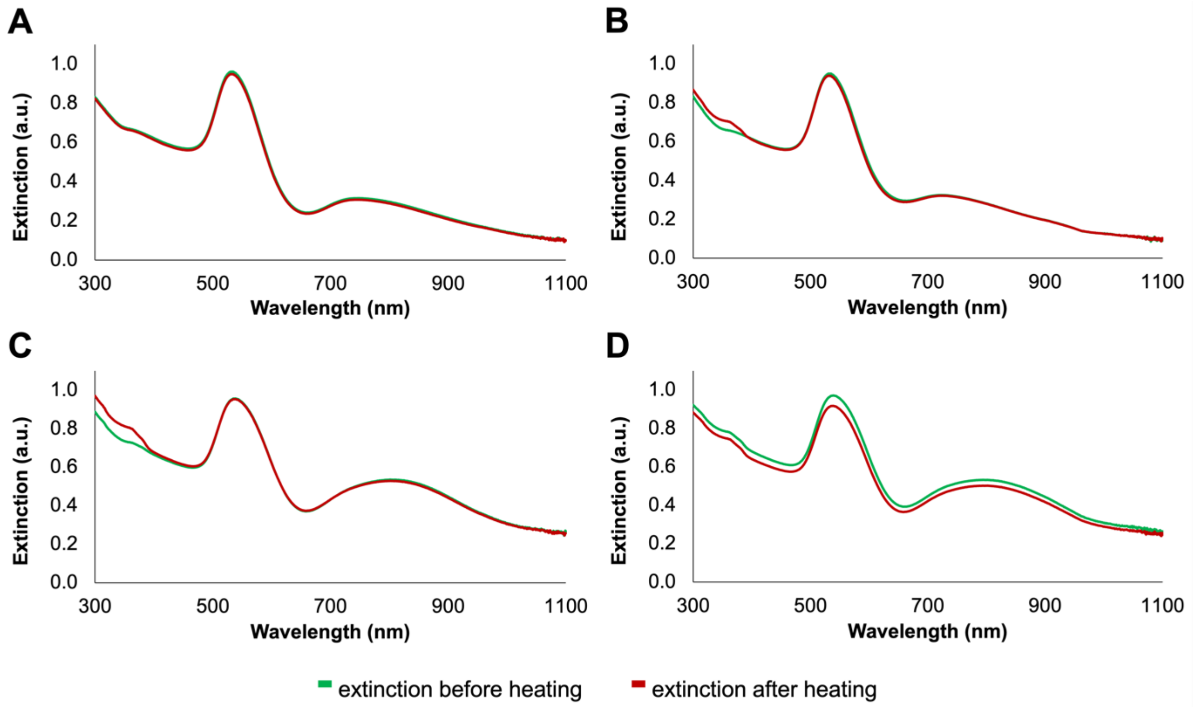

Figure S12: Extinction spectra before and after photothermal heating for (A) SHINs, (B) SHINs + TEOS, (C) silica-capped aggregates, and (D) silica-capped aggregates + TEOS, collected using a Cary-60 UV-vis spectrometer scanning from 300 nm to 1100 nm at a scanning rate of 600 nm/min. Samples were adjusted to an optical density of 1 for a volume of 500 µL.

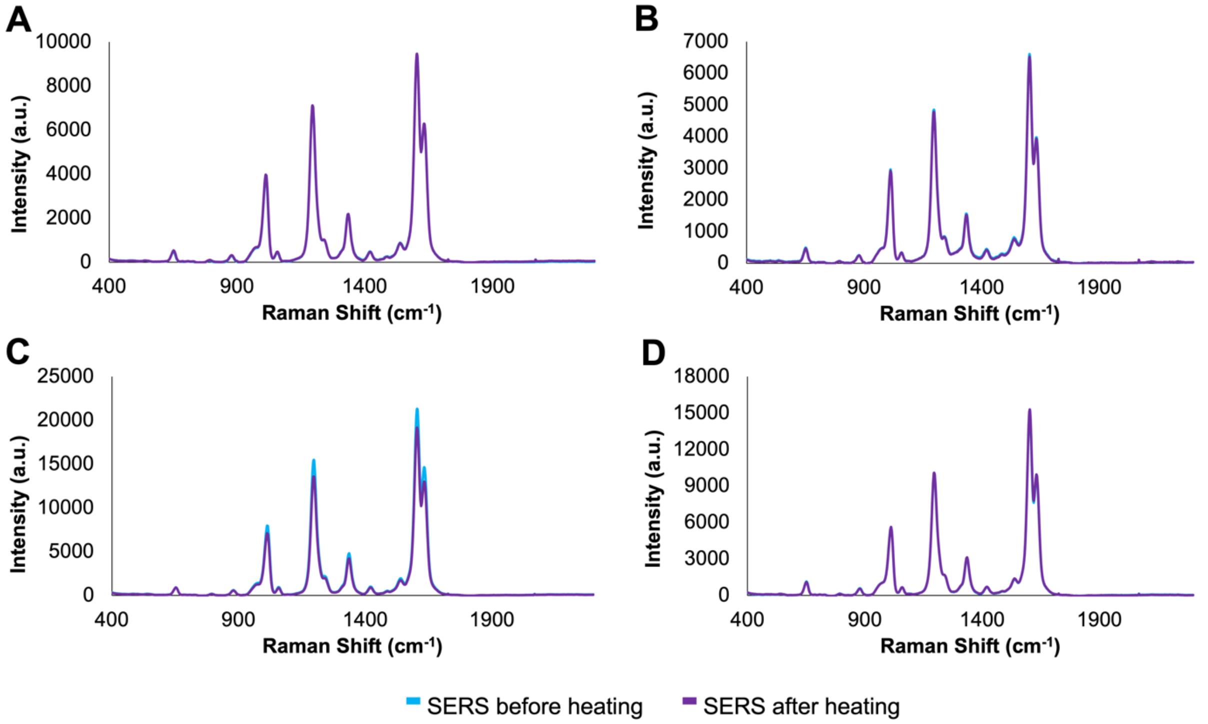

Figure S13: SERS spectra before and after photothermal heating for (A) SHINs, (B) SHINs + TEOS, (C) silica-capped aggregates, and (D) silica-capped aggregates + TEOS, collected using a Snowy Range Instruments CBex handheld spectrometer at an excitation wavelength of 785 nm, laser power of 10 mW, and acquisition time 0.1 s. SERS spectra were baseline corrected using MatLab (Version 2022b) and plotted in Excel. Samples were adjusted to an optical density of 1 for a volume of 500 µL.

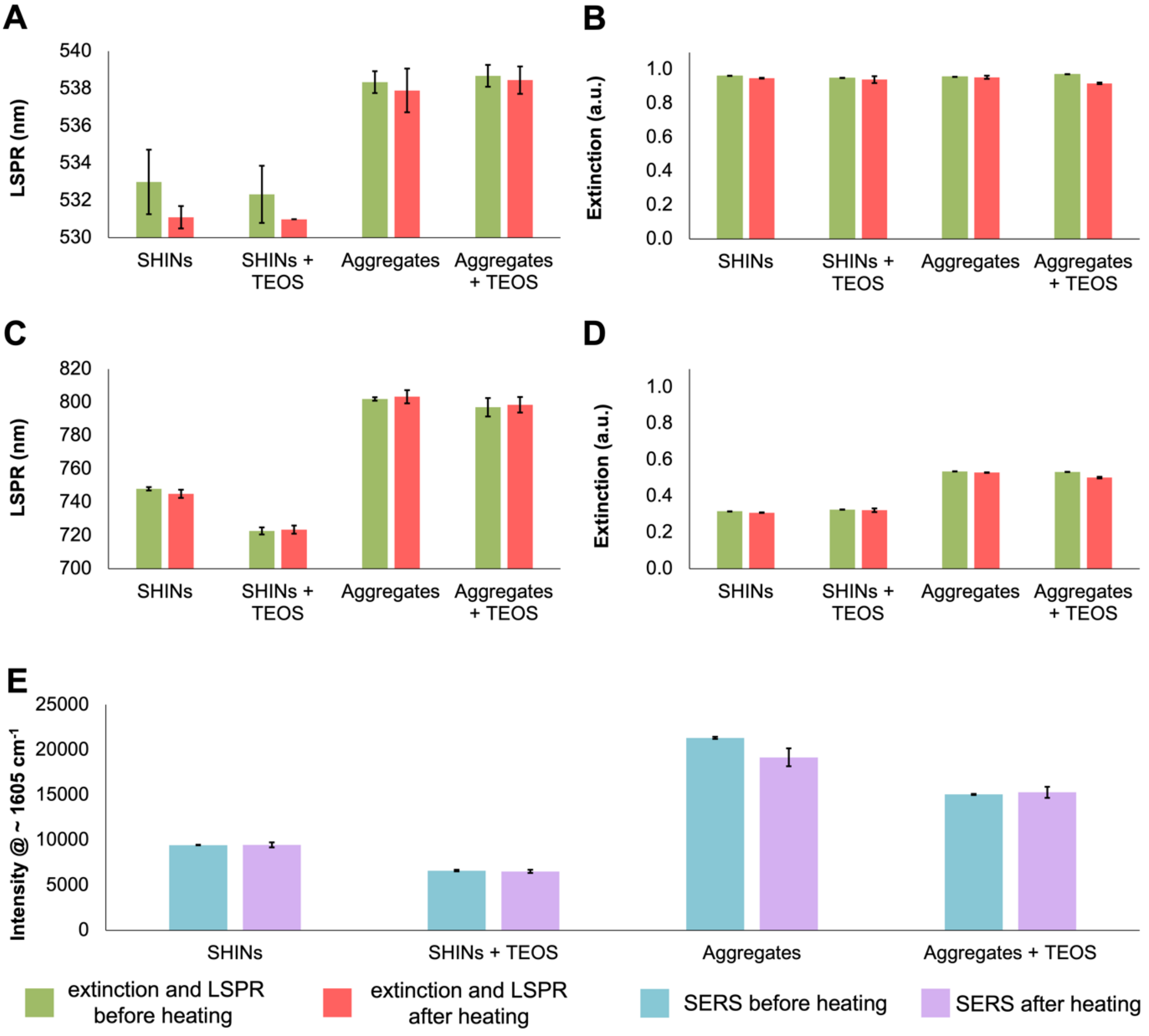

Figure S14. Changes in the (A) position and (B) extinction intensity of the LSPR in the visible region, and (C) position and (D) extinction intensity of the NIR LSPR, for SHINs and aggregates prepared with and without TEOS before and after photothermal heating. Extinction spectra were collected using a Cary60 UV-vis spectrophotometer scanning from 300 to 1100 nm at a medium scanning rate of 600 nm/min. (E) Changes in the BPE SERS peak intensity at ~ 1605 cm^{-1} for the same samples before and after heating. SERS spectra were collected using a handheld Snowy Range Instruments CBex spectrometer at an excitation wavelength of 785 nm, laser power of 10 mW at the sample, and acquisition time of 0.1 s. Following collection, spectra were baseline corrected using MatLab (Version 2022b). For characterisation with extinction spectroscopy and SERS, samples were adjusted to an optical density of 1 for a volume of 500 µL.
